# Forage Rotations Conserve Diversity of Arbuscular Mycorrhizal Fungi and Soil Fertility

**DOI:** 10.3389/fmicb.2019.02969

**Published:** 2020-01-09

**Authors:** Elisa Pellegrino, Hannes A. Gamper, Valentina Ciccolini, Laura Ercoli

**Affiliations:** ^1^Institute of Life Sciences, Scuola Superiore Sant’Anna, Pisa, Italy; ^2^SCL Italia SpA, Pisa, Italy

**Keywords:** arbuscular mycorrhizal fungi (AMF), community analysis, legume forage, land use, olive orchard, soil carbon, soil fertility, vineyard

## Abstract

In the Mediterranean, long-term impact of typical land uses on soil fertility have not been quantified yet on replicated mixed crop-livestock farms and considering the variability of soil texture. Here, we report the effects, after 15 years of practice, of two legume-winter cereal rotations, olive orchards and vineyards on microbiological and chemical indicators of soil fertility and the communities of arbuscular mycorrhizal fungi (AMF). We compare the changes among these four agricultural land-use types to woodland reference sites. Root colonization by AMF of English ryegrass (*Lolium perenne* L.), a grass that occurred under all land use types, was only half as heavy in biannual berseem clover (*Trifolium alexandrinum* L.)-winter cereal rotations than in 4-year alfalfa (*Medicago sativa* L.)-winter cereal rotations. In olive (*Olea europaea* L.) orchards and vineyards (*Vitis vinifera* L.), where weeds are controlled by frequent surface tillage, the AMF root colonization of ryegrass was again much lower than in the legume-cereal rotations and at the woodland reference sites. All the microbial parameters and soil organic carbon correlated most strongly with differences in occurrence and relative abundance (β-diversity) of AMF genera in soil. The soil pH and mineral nutrients in soil strongly correlated with differences in AMF root colonization and AMF genus richness (α-diversity) in soil. Diversity of AMF was much less affected by soil texture than land use, while the opposite was true for microbial and chemical soil fertility indicators. Land uses that guaranteed a continuous ground cover of herbaceous plants and that involved only infrequent tillage, such as multiyear alfalfa-winter cereal rotation, allowed members of the AMF genus *Scutellospora* to persist and remain abundant. On the contrary, under land uses accompanied by frequent tillage and hence discontinuous presence of herbaceous plants, such as tilled olive orchard and vineyard, members of the genus *Funneliformis* dominated. These results suggest that multiyear alfalfa-winter cereal rotation with active plant growth throughout the year is the least detrimental agricultural land use in soil carbon and AMF abundance and diversity, relative to the woodland reference.

## Introduction

Soil quality has been defined as “the capacity of a soil to function within ecosystem boundaries to sustain biological productivity, maintain environmental quality and promote plant and animal health” (Doran and Parkin, 1994). This definition captures the multi-functionality of agro-ecosystems, which provide biomass, food and fiber, store and filter water, and maintain biodiversity and biogeochemical cycles. In agroecosystems, where the main, though not exclusive, service of soils is the maintenance of crop yield, soil quality is best characterized by a combination of chemical and microbiological indicators of soil fertility (Bünemann et al., 2018; Schloter et al., 2018). This is because a fertile and thus productive soil has to supply plants with essential nutrients and water, be free of toxic substances, and support a diverse and active community of biota, which contribute to element cycling, nutrient retention, and soil structure (Power and Prasad, 1997; Mäder et al., 2002; Bedini et al., 2009). Therefore, it is key to adopt land use types and agricultural practices that maintain soil organic carbon (SOC) and microbial biomass, activity and diversity to guarantee a stable mineral nutrient supply to crops over the long-term (Jeffries et al., 2003; Oldfield et al., 2019).

Agricultural practices, such as use of cover crops, tillage, and application of organic and mineral fertilizers, modulate the amount of SOC and microbial abundance and activity, and increase the availability of nitrogen (N), potassium (K), phosphorus (P) and sulphur (S) in soil (Lu et al., 2011; Ercoli et al., 2012; Aguilera et al., 2013; Hallama et al., 2019). Intensive production usually reduces SOC due to reduced organic matter returns to soil, lower crop cover and frequent tillage. Therefore, most agricultural soils show considerably lower SOC contents than soils with a natural and continuous vegetation cover (Sanderman et al., 2017).

Mediterranean soils are known to be particularly sensitive to SOC degradation (Hernández et al., 2016) and to have a slower biogeochemical nutrient cycling and lower water retention than soils from more humid and cold regions (Guo and Gifford, 2002; Kassam et al., 2012; Aguilera et al., 2013). High temperatures throughout the year and frequent soil tillage enhance microbial decomposition of SOC, and summer droughts reduce plant growth and thus litter inputs to soil. Given the utmost importance of SOC for soil fertility and carbon (C) sequestration (Lal and Stewart, 2010; Oldfield et al., 2019), it is a matter of urgency to identify suitable agricultural land-use types and adopt measures to preserve SOC (Aguilera et al., 2013; Searchinger et al., 2018; Pretty, 2018; Oldfield et al., 2019). However, few studies have been carried out with the same land use types replicated at multiple sites to quantify the impact of Mediterranean land-use types on soil fertility indicators and soil biota involved in crop performance and soil quality (Armenise et al., 2013; Ciccolini et al., 2016b; Powell and Rillig, 2018).

One important group of soil biota that supports plants in nutrient and water uptake and contributes to soil ecosystem services, such as soil aggregation and C sequestration, is the arbuscular mycorrhizal fungi (AMF, phylum Glomeromycota, Xu et al., 2017; Powell and Rillig, 2018; Tedersoo et al., 2018). Arbuscular mycorrhizal fungi colonize the roots of most land plants, including the majority of crops, and exchange primarily plant-growth limiting mineral nutrients against photosynthates (Smith and Read, 2008). They increase plant access to patches of soil where nutrients are released (Joner and Jakobsen, 1995; Tibbett, 2000) and exploit pore space which is inaccessible to roots, thereby improving nutrient and water use of crops (Li et al., 2008; Cavagnaro et al., 2015; Ercoli et al., 2017; Manoharan et al., 2017; Mai et al., 2019).

Past studies have shown that AMF abundance and diversity can be severely reduced by intensive land use associated with application of mineral fertilizer and tillage (Jansa et al., 2002; Oehl et al., 2005; Brito et al., 2012; Xu et al., 2017). Tillage and crop identity have been shown to shape the composition and structure of the communities of AMF and other microbes (Helgason et al., 1998; Six et al., 2006; Ngosong et al., 2010; Pellegrino et al., 2014; Ciccolini et al., 2015, 2016b). Diversity and continuous presence of different AMF host plants, as it is the case in grasslands and when multispecies forage and cover and catch crops are grown, are known to maintain high AMF abundance and diversity (Oehl et al., 2005; Verbruggen et al., 2010; Creamer et al., 2016; Klabi et al., 2018; Higo et al., 2019; Schmidt et al., 2019) and to sustain C sequestration in soil (Six et al., 2006; Xu et al., 2017).

Preceding crops and residual effects of agronomic interventions cause legacies in chemical and biological soil properties (Hallama et al., 2019), including microbial biomass, AMF abundance and diversity (Ngosong et al., 2010; Brito et al., 2012; Detheridge et al., 2016; Klabi et al., 2018; Faggioli et al., 2019; Higo et al., 2019; Lehman et al., 2019) with cascading effects on crop productivity (Manoharan et al., 2017). Moreover, composition and structure of AMF communities are affected by soil texture, moisture and aeration (Lekberg et al., 2007). Accounting for confounding effects by differences in soil texture is thus important in field surveys to study the effects of land use on soil fertility at different locations, such as on different farms (Ciccolini et al., 2016a).

Different land uses act as an ecological filter in the assembly of AMF communities, since they determine the survival and propagation of AMF (Lekberg et al., 2007; Pellegrino et al., 2014; Ciccolini et al., 2016b). Particular land-use types determine the prevalence of AMF (i.e., the absolute abundance expressed as root colonization), the dominance of specific AMF taxa and the AMF diversity (i.e., the combination of richness and relative taxon abundance) within land-use types (α-diversity) as well as AMF community composition (i.e., taxon occurrence) and structure (i.e., relative taxon abundance), which may differ among sites (β-diversity). Agricultural soil management is also likely to homogenize the communities of AMF by dispersing and favoring ruderal and disturbance-tolerant taxa, and by reducing spatial heterogeneity in soil fertility and vegetation cover (Rosendahl et al., 2009; Chagnon et al., 2013; Pärtel et al., 2017; De León et al., 2018). Therefore, fast-growing, disturbance-tolerant and abundantly sporulating AMF taxa can be expected to become dominant (Chagnon et al., 2013; Xu et al., 2017). Moreover, the occurrence of tillage-sensitive taxa can be expected to become restricted to deeper soil layers, vegetation buffer strips, or to agricultural land-uses with less intensive tillage, such as multiyear forage (Oehl et al., 2005; Peyret-Guzzon et al., 2015; Sosa-Hernández et al., 2018).

In the Mediterranean climate, forage systems are characterized by dense and continuous ground cover and hence abundant and continuous fluxes of C to roots and AMF. Conversely, woody crops, such as olive trees and vineyards, show reduced C fluxes to the topsoil layer, because of necessary frequent surface tillage to minimize nutrient and water competition by weeds. Rarity and low abundance of AMF with long life cycles and limited spore production were already reported in another study in olive orchards (Montes-Borrego et al., 2014). Moreover, while abundant root litter and exudation under multiyear forage crops can be expected to replenish SOC (Six et al., 2006; Aguilera et al., 2013; Sokol et al., 2019), surface tillage under woody crops can be expected to promote the microbial degradation of SOC (Six et al., 2006; Schmidt et al., 2019). Residual mineral fertilizer not absorbed by crops is, furthermore, probable to promote the microbial degradation of SOC even further, particularly under conditions of relative N limitation (Pausch et al., 2016). Temporal P and K excess in the topsoil in absence of a dense ground cover of vigorously growing plants is, hence, likely promoting the decomposition of SOC and reducing C allocation to AMF. On the contrary, presence of large root masses that introduce C to soil and allow for C fluxes to AMF can be expected to support SOC formation and AMF abundance.

The present field study was conducted with the objective to fill the gap of information on Mediterranean land-use types with the intention to identify the agricultural land-use type(s) with the least negative impact on soil C, AMF diversity and soil fertility. We hypothesized that soil C, indicators of soil fertility and AMF abundance, richness and diversity decline with the increase of agricultural intensification (i.e., tillage, fertilization and ground cover). We hence tested for differences among land uses in chemical and microbiological soil properties, AMF abundance in roots and AMF composition and relative abundance (i.e., diversity) in soil. Moreover, we studied the direction and magnitude of change with respect to adjacent woodlands not under agricultural management. We further explored the relationship between AMF α- and β-diversity and soil properties and between AMF α- and β-diversity.

## Materials and Methods

### Study Area, Soil Type and Climate

The study area is located in the municipality of Manciano next to the town of Grosseto (42° 33′ N, 11° 26′ E; 306 m above sea level), a hilly, inland area in southern Tuscany in central Italy (Supplementary Figure S1), which had been covered by mixed oak (*Quercus* spp.) - ash (*Fraxinus* spp.) woodland until the 1950’s when it was converted for agricultural use. Today, 63% of the total land surface in the area is used for agriculture and only about 19% is covered by either relict or re-established woodlands (ISTAT, 2010). The agricultural land is mostly used for five crops, namely multiyear forage crops, such as alfalfa (*Medicago sativa* L., 4.3% of the total agricultural land) and oat (*Avena sativa* L.) - berseem clover (*Trifolium alexandrinum* L.) mixtures (52.1%) for forage production, olive orchards (*Olea europaea* L., 6.3%) or vineyards (*Vitis vinifera* L., 2.5%).

The soil of the area is a *Haplic Calcisol*, according to the FAO classification system (IUSS, 2015), and an *Inceptisol*, according to the USDA classification (Soil Survey Staff, 1999). Its texture ranges from clay to loam (Table 1). The climate is Mediterranean (Csa, according to the Köppen–Geiger climate classification) with dry and hot summers and most rainfall occurring in autumn and spring. The mean annual rainfall is 730 mm and the average monthly air temperature ranges between 4.7°C in January and 22.1°C in July (annual mean 13.1°C, Vallebona et al., 2015).

**TABLE 1:** Physical, chemical, and microbiological soil properties under five land-use types on three mixed crop-livestock farms in the municipality of Manciano (Tuscany, central Italy).

**Parameter^a^**	**Land-use type^b^**				
	**AA**	**OC**	**TO**	**TV**	**WO**
***Physical***					
Clay (%)	38.7 ± 1.2b^c^	43.2 ± 1.9b	34.3 ± 1.4b	40.6 ± 2.9b	16.4 ± 5.6a
Silt (%)	31.0 ± 1.2	38.3 ± 3.4	30.5 ± 1.9	35.9 ± 2.6	35.7 ± 2.3
Sand (%)	30.3 ± 1.7bc	18.5 ± 1.5a	35.2 ± 0.4c	23.5 ± 1.6ab	47.9 ± 5.0d
Texture class	Clay-loam	Clay	Clay-loam	Clay	Loam
***Chemical***					
pH (H_2_O)^d^	7.1 ± 0.1ab	8.1 ± 0.0b	7.7 ± 0.1ab	7.9 ± 0.1ab	6.2 ± 0.0a
K_exch_ (mg kg^–1^)	167.4 ± 6.3a	177.9 ± 24.2a	290.7 ± 3.5ab	223.0 ± 21.8ab	340.3 ± 53.0b
P_avail_ (mg kg^–1^)	9.62 ± 2.42ab	6.46 ± 1.72a	22.83 ± 6.40ab	36.17 ± 18.96b	7.53 ± 1.28ab
P_tot_ (mg kg^–1^)	824.4 ± 75.9a	1172.2 ± 34.8ab	1608.0 ± 78.9b	1667.0 ± 208.2b	918.3 ± 109.0a
N_tot_ (g kg^–1^)	1.48 ± 0.11a	1.43 ± 0.04a	3.10 ± 0.32b	1.52 ± 0.20a	5.12 ± 0.17c
SOC (g kg^–1^)	17.0 ± 1.6ab	10.6 ± 0.9a	26.9 ± 0.9b	11.1 ± 2.2a	68.9 ± 5.8c
C/N (mass-based)	11.5 ± 0.3*c*^d^	7.5 ± 0.7ab	8.8 ± 0.7b	7.2 ± 0.5a	13.4 ± 0.7d
CEC (meq 100 g)	28.8 ± 1.1c	22.9 ± 1.8ab	25.6 ± 0.9b	26.3 ± 1.9a	36.2 ± 3.8d
***Biochemical***					
SR (mg C kg^–1^ soil)	107.5 ± 4.1*b*^d^	102.3 ± 16.7ab	89.5 ± 2.2ab	142.3 ± 0.8c	81.7 ± 3.84a
MBC (mg C kg^–1^ soil)	186.7 ± 9.3ab	166.7 ± 27.9ab	143.7 ± 5.7a	148.3 ± 4.4a	216.3 ± 6.52b
qCO_2_ (mg CO_2_-C g^–1^ C_mic_ h^–1^)	0.58 ± 0.05b	0.64 ± 0.13b	0.62 ± 0.01b	0.96 ± 0.02c	0.38 ± 0.03a
C_mic_/C_org_ (%)	1.11 ± 0.08abc	1.61 ± 0.36c	0.53 ± 0.04ab	1.43 ± 0.26bc	0.32 ± 0.02a

*Values represent the means ± standard errors of composite soil samples taken at 0–30 cm soil depth from each field. ^*a*^K_*exch*_, exchangeable potassium; P_*avail*_, available phosphorus; P_*tot*_, total phosphorus; N_*tot*_, total nitrogen; SOC, soil organic carbon; CEC, cation exchange capacity; SR, soil respiration; MBC, microbial biomass carbon; qCO_2_, metabolic quotient; C_*mic*_/C_*org*_, microbial carbon to soil organic carbon ratio. ^*b*^AA, 5-year rotation with four years of alfalfa (*Medicago sativa* L.) and one year of winter cereal [durum wheat (*Triticum turgidum* L. subsp. *durum* (Desf.) Husn.) or *triticale* (×Triticosecale Wittm. ex A. Camus)]; OC, 3 years rotation with two years of oat (*Avena sativa* L.) – berseem clover (*Trifolium alexandrinum* L.) mixture and one year of winter cereal (durum wheat or triticale); TO, tilled olive orchard (*Olea europea* L.); TV, tilled vineyard (*Vitis vinifera* L.); WO, woodland dominated by *Quercus cerris* L. and *Fraxinus ornus* L. ^*c*^Values for a variable followed by different letters are statistically different, according to the Tukey test (*P* ≤ 0.05). Data were analyzed by the analysis of variance (ANOVA) according to the randomized block design and using the percentage of clay and sand as covariables. ^*d*^Mean comparisons of the parameters C/N and SR are based on the Dunn non-parametric test (*P* ≤ 0.05).*

### Study Design

The study focused on five common land-use types that are typically found on the same mixed crop-livestock farm in the study area, namely 5-year rotation with 4 years of alfalfa (*Medicago sativa* L.) and 1 year of a winter cereal [durum wheat (*Triticum turgidum* L. *subsp. durum* (Desf.) Husn.) or triticale (× *Triticosecale* Wittm. ex A. Camus)] (AA), 3-year rotation with 2 years of oat (*Avena sativa* L.) - berseem clover (*Trifolium alexandrinum* L.) mixture and 1 year of a winter cereal (durum wheat or triticale) (OC), tilled olive orchard (*Olea europaea* L.) (TO), tilled vineyard (*Vitis vinifera* L.) (TV), and woodland (WO). The WO sites were chosen as pre-agricultural vegetation type and they were open-canopy woodlands dominated by *Quercus cerris* L. and *Fraxinus ornus* L. with a herbaceous ground cover.

Three farms were chosen that had been hosting all these five land-use types for at least 15 years on the same field before sampling, resulting in a randomized block design with a total of 15 study units (Supplementary Figure S1 and Supplementary Table S1). The three farms were treated as the blocks. The largest distance among any sampled field on any of the farms was 1.6 km and the smallest 60 m (Supplementary Figure S1 and Supplementary Table S1). Except for two fields on the largest farm, the distances between fields on the same farm were smaller than the distances between fields among farms. Detailed information about the applied crop management practices for the different land-use types can be found in the Supplementary Text S1.

### Root and Soil Sampling

The root systems of five English ryegrass (*Lolium perenne* L.) plants and five soil cores of 5 cm diameter and 30 cm depth, corresponding to the tilled and fertilized soil layer, were taken and pooled to obtain a composite root and soil sample of each field. The colonization of the roots of ryegrass by AMF was used as an indicator of AMF community abundance as influenced by land use (crop plant identity and agricultural management). The AMF in the soil samples were used to determine the influence of land use, i.e., crop plant identity and agricultural management on AMF diversity. English ryegrass was a suitable plant to determine the infection pressure of the soil-indigenous AMF, since it occurred under all five investigated land use types and had previously been used as a generalist host plant to trap AMF in pot cultures (Oehl et al., 2003). The root and soil sampling was done at the same time in April 2012 after the onset of plant growth, when the soil microflora and fauna are sufficiently active and abundant to reliably detect differences among land use types, as well as, to avoid direct influences by tillage and fertilization (Picci and Nannipieri, 2002). In case of the woody crops (olive and vine), the samples were taken in the interrow.

The roots were rinsed thoroughly after collection and only fine roots (<1 mm) were used to measure colonization by AMF (see below). The soil samples were immediately sieved to 2 mm and stored at 4°C before the microbiological analyses and the extraction of the total DNA. The chemical and textural analyses were done after air-drying.

### Soil Textural, Chemical and Microbiological Analyses

The soil samples were analyzed for texture by the hydrometric method (Gee and Bauder, 1986), pH in water (1:2.5 w/v), exchangeable potassium (K_exch_) with 1 M KCl (1:10 w/v, 30 min shaking), and total nitrogen (N_tot_) by Kjeldahl digestion (Bremner and Mulvaney, 1982). The SOC was determined using the modified Walkley–Black wet combustion method (Nelson and Sommers, 1982). The total phosphorus (P_tot_) was extracted with perchloric acid and the bioavailable phosphate (P_avail_) was extracted alkaline sodium bicarbonate following Olsen and Sommers (1982) and measured by colorimetry (Ohno and Zibilske, 1991). The cation exchange capacity (CEC) at pH 8.1 was determined by displacement with 0.1 M BaCl_2_ triethanolamine (Hendershot and Duquette, 1986). The C/N ratio was calculated as the ratio of SOC and N_tot_ (Jagadamma et al., 2007). The microbial biomass carbon (MBC) and soil respiration (SR) were assessed in soil samples whose humidity had been adjusted to 60% of field capacity. The MBC was determined in 60 g of soil, using chloroform fumigation for 5 days at 20°C (Vance et al., 1987). The SR was estimated in 45 g of soil incubated at 30°C in the dark in closed glass jars, according to the Isermeyer method (Alef and Nannipieri, 1995), which involves trapping the evolved CO_2_ with 0.5 N NaOH over the course of 5 days of incubation and determining the NaOH excess by titration with 0.1 N HCl. The microbial carbon (C_mic_) to total carbon (C_mic_/C_org_) ratio and metabolic quotient (qCO_2_), calculated as the SR/MBC ratio, were used as indicators of the microbial contributions to SOC and SR, respectively (Insam and Haselwandter, 1989; Anderson and Domsch, 1990).

### Arbuscular Mycorrhizal Fungal Abundance in Roots

The roots of English ryegrass were cleared and the AMF structures stained for microscopic quantification of the percentage of root colonization, using the method of Phillips and Hayman (1970), except that the phenol was replaced with lactic acid. The fraction of roots colonized by AMF was determined under the dissecting microscope (Olympus SZX 9, Olympus Optics, Tokyo, Japan), using gridline intersection counting of the colonized and not colonized root sections (Giovannetti and Mosse, 1980). To verify that AMF and not mistakenly other fungi or staining artifacts were quantified, some root fragments were mounted on microscopic slides and examined in detail at 125-500-1250 × magnification for characteristic features of AMF, such as thick and aseptated hyphae of unequal diameter filled with oil globules.

### DNA Extraction, PCR Amplification and 18S rRNA Partial Gene Sequencing

Total DNA was extracted from approximately 0.5 g of dry soil, using the PowerSoil^®^ MoBio extraction kit (Mo Bio Laboratories Inc., New York, NY, United States). The purity and approximate concentration of the extracts were measured using a ND-1000 spectrophotometer (NanoDrop Technology, Wilmington, DE, United States). A fragment of the nuclear ribosomal small subunit gene (18S rRNA) was amplified by polymerase chain reaction (PCR), using the primer pair NS31 and AM1 (Simon et al., 1992; Helgason et al., 1998). In brief, 10 ng μl^–1^ of genomic DNA template were used in reactions of 20 μl, using 0.5 U of GoTaq^®^ Hot Start Taq polymerase (Promega Corporation, Madison, WA, United States), 0.2 μM of each primer (NS31/AM1), 0.2 mM of each dNTP, 1.25 mM of MgCl_2_ and 1x reaction buffer in a S1000 Thermal Cycler^TM^ (Bio-Rad, Hercules, CA, United States). The PCR amplicons were column-purified with the QIAquick PCR purification kit (Qiagen, Hilden, Germany), quantified using a ND-1000 spectrophotometer, and ligated into the pGem^®^-T Easy vector (Promega Corporation, Madison, WA, United States), which was used to transform XL10-Gold^®^ Ultracompetent *Escherichia coli* cells (Stratagene^®^, La Jolla, CA, United States) to multiply it clonally. At least 25 recombinant clones per amplicon library, which corresponded to a field soil sample, were screened by PCR reamplification for the approximately 550 bp-long NS31/AM1 insert. Inserts of appropriate size were Sanger sequenced on an ABI^®^ Prism 3730XL automated capillary sequencer (Applied Biosystem, Foster City, CA, United States) from plasmid DNA extracted from miniprep liquid cultures at the High-Throughput Genomics Unit (Seattle, WA, United States), using vector primer SP6.

### Phylogenetic Sequence Analysis and Inference of Taxonomic and Phylogenetic Diversity

**FIGURE 1:**
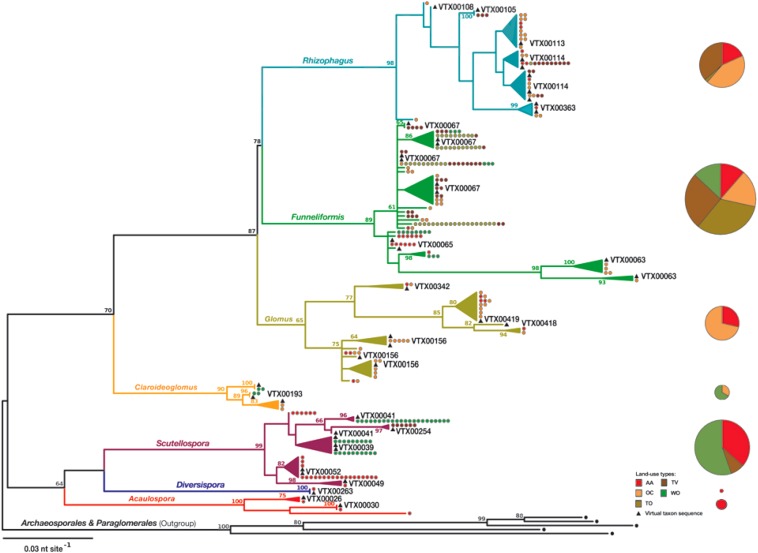
Maximum likelihood (ML) tree to phylogenetically assign the new partial 18S rRNA gene sequences from soil to seven genera of arbuscular mycorrhiza fungi (AMF, phylum Glomeromycota) and accession sequences of *Virtual Taxa* (VT) of the *MaarjAM* database (Öpik et al., 2010). The absolute abundance (pie diameter) and relative abundance of the genera in five land-use types (size of pie slices) is indicated at the right [AA, 5 years rotations with 4 years of alfalfa (*Medicago sativa* L.)] and 1 year of a winter cereal [durum wheat (*Triticum turgidum* L. *subsp. durum* (Desf.) Husn.) or triticale (× *Triticosecale* Wittm. ex A. Camus)]; OC, 3-year rotation with 2 years of oat (*Avena sativa* L.) – berseem clover (*Trifolium alexandrinum* L.) mixture and 1 year of winter cereal (durum wheat or triticale); TO, tilled olive (*Olea europea* L.) orchards; TV, tilled vineyards (*Vitis vinifera* L.); WO, woodlands dominated by *Quercus cerris* L. and *Fraxinus ornus* L. The tree (GAMMA-based ML: −4051.61) was constructed with 279 new partial 18S rDNA gene sequences (550 bp, accession numbers: LN714838-LN715116) and phylogenetically closest accessions of VT reference sequences of >97% identity. The tree is rooted with representative sequences of the five genera of phylogenetically basal AMF orders Archaeosporales and Paraglomerales. Bootstrap values > 60% are shown at the internal nodes of the tree. The number of identical sequences per representative sequence is indicated as dots, whose colors refer to the land-use type from which they were recovered. See Supplementary Figure S2 and Supplementary Table S2 for the accession numbers of the representative and identical sequences and the accession numbers of the phylogenetically closest accessions of VT.

Two hundred and seventy nine newly generated partial 18S rRNA gene sequences, showing similarities to AMF in *BLASTn* searches against the public sequence databases, were aligned with *MUSCL* version 3.8.31 in *SeaView* version 4.5.4. Then, after excluding identical sequences from the multiple alignment, a maximum likelihood (ML) tree was calculated in *RAxML* version 8.2.10 on CIPRES Science Gateway version 3.3. Corresponding Virtual Taxon (VT) reference sequences (>97% sequence identity, Öpik et al., 2010), as identified in *BLASTn* similarity searches, were downloaded from the *MaarjAM* sequence database and included into ML trees inferred by the *GTR* + *GAMMA* sequence evolutionary model. The final ML tree, for which the most phylogenetically related VT reference sequence was chosen for each clade of new sequences, was rooted with representative sequences of each of the genera of the phylogenetically basal orders of AMF, the Archaesporales and Paraglomerales (Figure 1).

The ML analysis considered 217 informative sites after manual optimization of the multiple sequence alignment and exclusion of potentially misaligned indel sites, using *Mesquit* version 3.51. Branch support values were obtained by *Rapid Bootstrapping*, using 1000 resampled datasets. To calculate weighted UniFrac distances and Faith’s Phylogenetic Diversity (PD) (see below) another ML tree was inferred in *RAxML* with only the new sequences, after exclusion of all reference sequences. The presented ML tree was edited in *FigTree* version 1.4.4 and further annotated in *Inkscape* version 0.93. Bootstrap branch support values >60% were retained and shown at the internal nodes of the tree (Krüger et al., 2012). All the sequences were deposited in the nucleotide sequence database of the European Molecular Biology Laboratory (EMBL) under the accession numbers LN714838-LN715116. Detailed information about the raw sequence data and taxonomic coverage can be found in the Supplementary Text S2.

The taxonomic diversity of the AMF communities in the soil of each sampled field and among the land-use types were analyzed at the genus level, which are monophyletic clades and whose members thus may share traits. This level of phylogenetic resolution was chosen to ensure that the sequencing effort was sufficient for adequate community coverage for the statistical analyses. The adequacy of the chosen effort of clone library sequencing and level of taxonomic resolution was verified by rarefaction analysis in *Analytic Rarefaction* version 2.0 (Supplementary Figure S2). Genus richness (#AMF), dominance (D) and Shannon diversity (H) were determined as components of α-diversity in *PAST* version 3.20 and β-diversity in the form of Bray-Curtis distances was calculated from the square roots of the number of sequences per genus (Supplementary Table S2). The PD was calculated as the weighted UniFrac distances in *mothur* version 1.40.5, using the ML tree of only the sequences of this study and the identical sequences as a measure of relative abundance (Supplementary Code S1). An indicator taxon analysis for the land-use types was run in R package *labdsv* version 1.8.0, using the *indval* function and as proposed by Dufrene and Legendre (1997) (Supplementary Code S2).

### Uni- and Multivariate Statistical Analyses

All measured microbiological and chemical soil properties, the fractional root length of English ryegrass colonized by AMF (%AMF), and the taxonomic α-diversity parameters (#AMF, D, H) were tested for possible influence of land use, using analysis of variance (ANOVA), according to the study design with farm as a blocking factor (i.e., ‘land-use type’ nested in ‘farm’), in the case that the residuals were normally distributed and the variances homogeneous, and, using non-parametric Kruskal–Wallis tests, in the case that the ANOVA assumptions were not fulfilled, even after Box–Cox transformation of the data. In case of a significant overall effect by the factor land use, the means for all land-use types were further compared by Tukey–Honestly Significant Difference, or Dunn’s non-parametric multiple comparison tests, respectively. Clay, silt and sand were used as covariables, since these are site-inherent mediating parameters, not affected by land use. All univariate statistical analyses were carried out in *JMP* version 14.1.0 (SAS, Institute Inc., Cary, NC, United States).

The phylogenetic divergence and taxonomic differentiation of the AMF communities (β-diversity) and distinctiveness of the soils with respect to microbiological and chemical properties under the different land uses were visualized by non-metric multidimensional scaling (NMDS) of the UniFrac, Bray–Curtis of the AMF communities and Euclidean distances of the microbiological and chemical soil properties. The analyses were performed in the software *PAST*. The AMF diversity data was set in relation to all those soil microbiological and chemical parameters found to be significantly affected by land use (Table 1), in an attempt to identify possible indirect effects, i.e., effects contingent on changes in soil properties and not direct effects of land use or agricultural practices on the AMF communities. To identify the land-use types, soil microbiological and chemical properties and components of AMF α-diversity that could have structured the AMF communities, the Bray–Curtis distances calculated from the square root-transformed AMF genus abundances were analyzed by constrained ordination analysis (Redundancy Analysis, RDA) with forward selection and unrestricted Monte Carlo permutation. The soil clay and sand contents, characterizing the sampling sites, were used as covariables. Since only few parameters were found to structure the AMF communities, also a Principle Component Analysis (PCA) was run to highlight the correlative relationships and relative importance of all parameters. For these analyses and those mentioned below, the microbiological and chemical soil properties and components of AMF α-diversity were rescaled to 0–1. The RDA and PCA were run in *CANOCO* for Windows version 5.11.

To further test the effect of land use and soil texture on AMF α, β-, and PD and the soil microbiological and chemical properties, permutational multivariate analyses of variance (PERMANOVA) were carried out, using the soil clay and sand contents as covariables and the farms as blocks (random factor) to account for the study design. The effect sizes were quantified by variance partitioning. The assumption of homogenous data dispersion among land-use types was verified by permutation testing (PERMDISP). These analyses were carried out with *PRIMER* version 6.1.15, using the add-on *PERMANOVA*+ version 1.0.3. The simultaneous effect of land use on (i) AMF β-diversity and soil microbiological and chemical properties, (ii) AMF α-diversity and soil microbiological and chemical properties, and (iii) AMF α- and β-diversity was analyzed by Partial Least Square (PLS) analyses in the software *PAST*. By considering the direction and proportional contributions (axis loadings) of the measured parameters, PLS analyses can reveal complex additive and subtractive effects among a multitude of measured parameters in multivariate space along the axes of greatest resolution. This analytical approach was thus considered ideal to reveal simultaneous land-use effects on soil microbiological and chemical properties as well as on AMF communities, which are key aspects of abiotic and biotic soil fertility, but whose correlation is not yet much explored.

## Results

### Soil Physico-Chemical and Microbiological Properties

Soil texture of the four agricultural land-use types was clay or clay-loam, while it was loam in WO (Table 1). The soil pH was lowest (slightly acidic) under WO, highest under OC (moderately alkaline) and neutral under the other land-use types (AA, TO, and TV). The bioavailable potassium (K) and phosphorus (P) concentrations were highest under TO and TV and total nitrogen (N) under WO, where SOC was also highest. The CEC was highest under WO sites and also higher under AA than under all other agricultural land-use types. SR was higher under TV and lowest under WO (Table 1). MBC was higher in the soil of the WO sites than in the intensively surface-tilled TO and TV. The metabolic quotient (qCO_2_) was lower in the soils of the WO sites than in the soils under agricultural land use. The microbial carbon to total organic carbon ratio (C_mic_/C_org_) was highest in the soil under OC and lowest in the soils under WO.

Across all land-use types, K_exch_ negatively correlated with pH (*R*^2^ = 0.27, *P* < 0.05). Total N positively correlated with SOC (*R*^2^ = 0.92, *P* < 0.001) and MBC negatively with pH (*R*^2^ = 0.51, *P* < 0.01). The qCO_2_ positively correlated with pH (*R*^2^ = 0.45, *P* < 0.001) as did C_mic_/C_org_ with SR (*R*^2^ = 0.48, *P* < 0.01).

### AMF Abundance in Roots and AMF Community Composition and Structure in Soil

**FIGURE 2:**
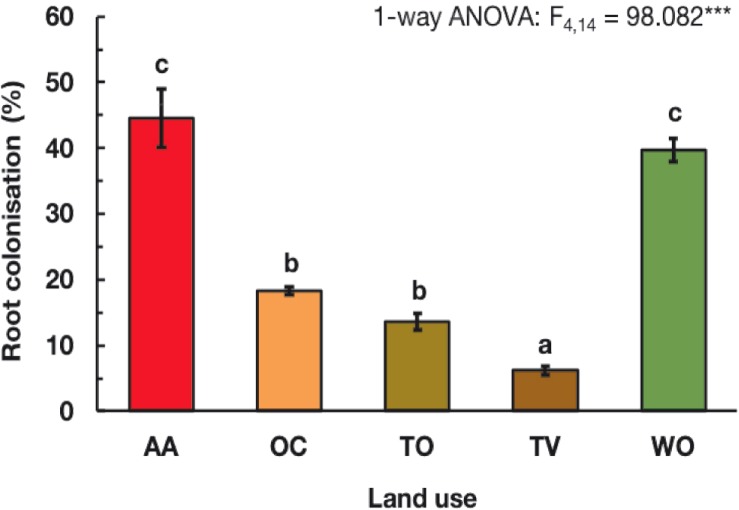
Arbuscular mycorrhizal fungal root colonization (%) of English ryegrass (*Lolium perenne* L.) under five land-use types. See Figure 1 for land-use abbreviations. Each bar represents the mean value ± SE of three fields on different farms per land-use type. Different letters above the bars indicate statistically significant differences, according to the Tukey test at *P* ≤ 0.05, following a one-way analysis of variance (ANOVA).

The colonization of the roots of English ryegrass by AMF varied more than sevenfold among the studied agricultural land-use types. It was under all, except one agricultural land-use type, considerably lower than at the non-agricultural woodland reference sites (WO) (Figure 2). The roots were colonized to approximately 45% of root length under AA, similar as at the WO reference sites. Under OC and TO, the colonization was on average only 16% and under TV even only 6%.

Clone library sequencing yielded 279 new partial 18S rRNA gene sequences of AMF from the soil samples (Supplementary Tables S2, S3). Rarefaction analyses at the genus level confirmed that the sequencing effort was sufficient for analyses of land use impacts on AMF diversity at this taxonomic resolution (Supplementary Figure S3).

Most AMF genera were recovered from soil at sites under AA, an intermediate number from the soils at sites used for OC, TV and WO, and least from the soil of TO (Supplementary Figure S4a), which was also reflected in the Shannon diversity (H), which followed a similar pattern (Supplementary Figure S4b). The dominance among the recorded AMF genera was highest in TO, intermediate in OC, TV, and WO, and lowest in AA (Supplementary Figure S4c). The PD of the AMF communities followed the Shannon diversity (H) (Supplementary Figure S4d).

Two AMF genera *Acaulospora* and *Diversispora* that were low in abundance were unique to AA (Figure 1 and Supplementary Figures S2, S5). Members of the genus *Funneliformis* were generally abundant and found in the soils of all land-use types. The genus *Funneliformis* was most dominant in TO and TV. Members of the genus *Rhizophagus* were found in all land-use types, except WO, but never reached dominance (Supplementary Figure S5). Members of *Scutellospora* were found in the soils under AA and TV and at the WO sites. The genus *Scutellospora* was dominant in WO. Members of the form genus *Glomus* occurred in the soils under AA and OC. The three genera *Funneliformis*, *Glomus*, and *Rhizophagus* were all about equally abundant in OC. Members of the genus *Claroideoglomus* were only found under OC and at the WO sites. The occurrence and prevalence of all seven AMF genera was, however, not sufficiently distinct and field replication not sufficient to recognize indicator taxa with statistical support (data not shown).

### AMF Communities and Soil Fertility Indicators Within and Among Land Use Types and Correlations Among Them

**FIGURE 3:**
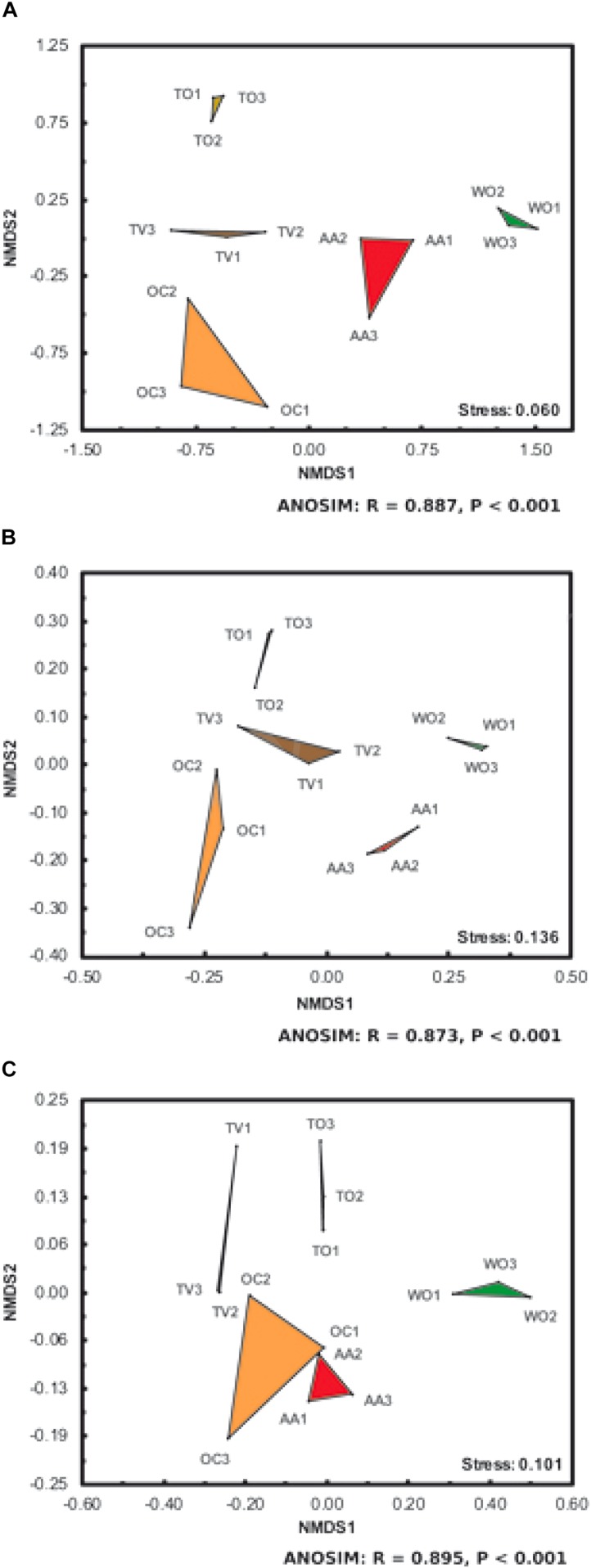
Non-metric multidimensional scaling (NMDS) of **(A)** phylogenetic dissimilarities and **(B)** genus-level structural differences of arbuscular mycorrhizal fungal (AMF) communities (β-diversity), and **(C)** differences in soil microbiological and chemical properties under five land-use types. See Figure 1 for land-use abbreviations. Each land-use type is represented by three fields on different farms (Supplementary Figure S1). The corresponding stress values and results of analyses of similarity (ANOSIM) are listed at the bottom right of each subfigure.

The AMF communities and soil properties at the WO sites were very distinct from those at the sites under agricultural land use (Figures 3A–C). The AMF communities and soil properties under agricultural land uses associated with frequent surface tillage (TO and TV) were furthermore distinct from those with a continuously present and active herbaceous ground cover (AA and OC), the forage crops. The microbiological and chemical soil properties between the land uses OC and AA were more distinct (Figure 3C) than the AMF communities (Figures 3A,B), which is graphically visualized by the NMDS plots (stress values < 0.136) and statistically supported (ANOSIM, *P* < 0.001).

Across all land-use types, the composition and structure of the AMF communities was best explained by differences in soil pH, AMF genus richness and the land use OC, according to a RDA (Supplementary Figure S6a). Many other soil parameters, AMF α-diversity parameters, and land-use types, however, correlated strongly with these three statistically influential variables, as evident from the PCA plot (Supplementary Figure S6b). According to this PCA, the occurrence and abundance of the genus *Scutellospora* was negatively correlated with soil pH (Supplementary Figure S6a). The occurrence and abundance of the genera *Claroideoglomus*, *Glomus*, and *Rhizophagus* were associated with the land-use type OC (Supplementary Figure S6b), whose soils are characterized by a high C_mic_/C_org_ ratio. The occurrence and abundance of the genera *Acaulospora*, *Diversispora*, and *Scutellospora* correlated with the land-use type AA, whose soils were characterized by high soil C/N, SOC, CEC, and %AMF. The prevalence of the ubiquitous, abundant and usually dominant genus *Funneliformis* (Supplementary Figure S5) was positively correlated with the availabilities of NPK (Supplementary Figure S6b), which were high in the soil in TO (Table 1).

Permutational multivariate analyses (PERMANOVA) revealed significant and quantitatively stronger effects on the composition and structure of the AMF communities by land use than soil texture (clay and sand content) (Table 2). Land use explained 47 and 49% of the total variance in the phylogenetic and taxonomic divergence of the AMF communities (β-diversity), respectively, while clay and sand content explained 40 and 39% of the variance in these two aspects of community differentiation, respectively. Likewise, land use and soil texture explained 64 and 25% of total variance in AMF α-diversity, respectively. On the contrary, the variance in the soil microbiological and chemical properties was better explained by soil texture than land use. Soil texture explained 52% and land use 32% of the total variance in the soil properties. Differences in AMF α- and β-diversity were better explained by the sand content of the soil than the clay content. The clay content, however, explained the differences in the soil microbiological and chemical properties better than the sand content.

**TABLE 2:** Permutational multivariate analyses of variance to test the effect of five land-use types on the composition and structure of arbuscular mycorrhizal fungal (AMF) communities among land-use types (*β*-diversity) and within land-use type (*α*-diversity), phylogenetic diversity (PD) and microbiological and chemical soil properties.

	**df**	**Pseudo-F**	**P(perm)**	**Explained variance (%)**	**PERMDISP**
***β-diversity (occurrence and abundance of AMF genera)***					
**Factors**					
Land use	4	10.180	0.001	46.9	*F*_4,10_ = 6.264^a^
Farm_rand_	2	3.090	0.062	4.4	
**Covariables**					
Clay (%)	1	25.514	0.004	15.8	
Sand (%)	1	9.141	0.017	23.9	
Residual	6			9.0	
Total	14				
***Phylogenetic diversity (PD, based on UniFrac distances)***					
**Factors**					
Land use	4	9.170	0.002	49.4	*F*_4,10_ = 11.852^a^
Farm_rand_	2	1.120	0.380	0.3	
**Covariables**					
Clay (%)	1	26.794	0.001	20.4	
Sand (%)	1	6.495	0.003	18.1	
Residual	6			11.8	
Total	14				
***α-diversity (%AMF, #AMF genera, D, H, PD)*^b^**					
**Factors**					
Land use	4	11.867	0.001	63.9	*F*_4,10_ = 2.688^a^
Farm_rand_	2	1.049	0.417	0.1	
**Covariables**					
Clay (%)	1	8.778	0.009	6.0	
Sand (%)	1	6.778	0.012	18.5	
Residual	6			11.5	
Total	14				
***Soil microbiological and chemical properties***					
**Factors**					
Land use	4	1.600	0.001	31.6	*F*_4.10_ = 9.776^a^
Farm_rand_	2	5.177	0.159	2.0	
**Covariables**					
Clay (%)	1	36.687	0.001	34.9	
Sand (%)	1	5.121	0.003	17.2	
Residual	6			14.4	
Total	14				

*Each land-use type was replicated on three farms, which were treated as a random factor according to the randomized block design. The percentages of clay and sand were used as covariables. The percentage of variance explained by land use and farm and covariables and the test of permutational analysis of multivariate dispersion (PERMDISP) are also shown. ^*a*^*P* > 0.05. ^*b*^ %AMF, percentage of root length of English ryegrass (*Lolium perenne* L.) colonized by AMF; #AMF, number of AMF genera; D, dominance of AMF at the genus level; H, Shannon diversity of AMF at the genus level; PD, phylogenetic diversity of AMF.*

**FIGURE 4:**
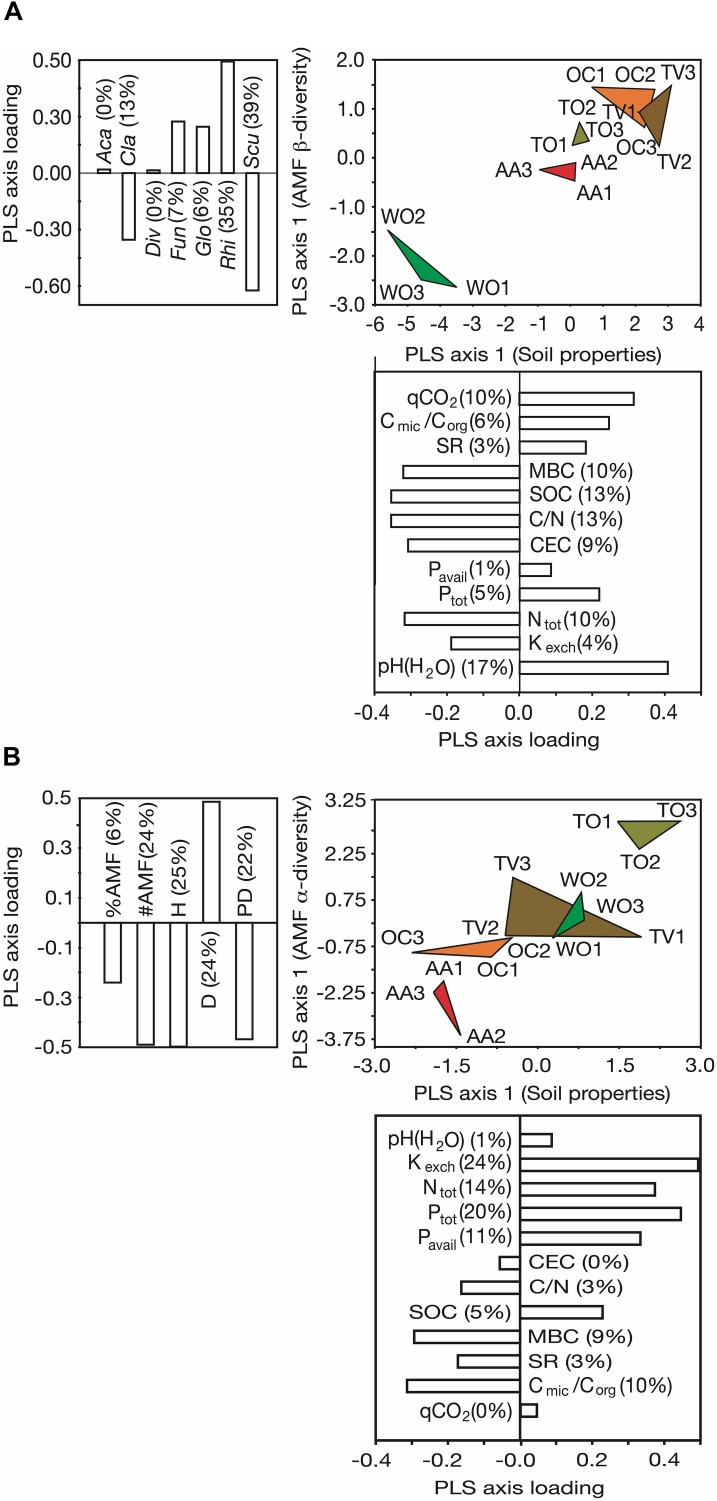
Partial least square (PLS) ordination plots separating five land-use types along the first ordination axes of soil microbiological and chemical properties and α- and β-diversity of arbuscular mycorrhizal fungal (AMF) communities at the genus level. **(A)** Ordination along the PLS axes of AMF β-diversity and soil microbiological and chemical properties and **(B)** ordination along the PLS axes of AMF α-diversity and soil microbiological and chemical properties. Three fields on different farms were analyzed per land-use type. The magnitude of influence (PLS axis loading) of the explanatory variables is indicated in the form of bar plots next to the ordination axes. The percentage contribution of the individual variables to the variance resolved by the respective ordination axis is indicated in brackets. See Figure 1 for land-use abbreviations. The AMF genera were: *Aca*, *Acaulospora* Gerd. and Trappe; *Cla*, *Claroideoglomus* C. Walker and A. Schüßler; *Div*, *Diversispora* C. Walker and A. Schüßler; *Fun*, *Funneliformis* C. Walker and A. Schüßler; *Glo*, *Glomus* Tul. and C. Tul.; *Rhi*, *Rhizophagus* P.A. Dang; *Scut*, *Scutellospora* C. Walker and F.E. Sanders. #AMF, number of AMF genera; %AMF, percentage of root colonization by AMF; CEC, cation exchange capacity; C_mic_/C_org_, microbial:soil organic carbon ratio; C/N, carbon:nitrogen ratio; D, dominance of AMF at the genus level; H, Shannon diversity of AMF at the genus level; K_exch_, exchangeable potassium; N_tot_, total nitrogen; PD, phylogenetic diversity of AMF; P_tot_, total phosphorus; P_avail_, bioavailable P; SOC, soil organic carbon; MBC, microbial carbon; qCO_2_, soil metabolic quotient; SR, soil respiration.

Joint ordination of the sampling sites along the axes of highest resolution through variation in AMF α- and β-diversity and soil microbiological and chemical properties (Figure 4) revealed strong and divergent impacts of agricultural land use on these aspects of abiotic and biotic soil fertility. The different land uses affected, both, the AMF β-diversity and soil microbiological and chemical properties, leaving behind distinctive multivariate legacies (Figure 4A). The legume-cereal forage rotation with 4 years of alfalfa (AA) was, notably, the least disruptive land-use type with reference to the WO sites, as evident by the smallest distance between AA and WO and its separation from other agricultural land-use types in the PLS plot. Increases in occurrence and abundance of the AMF genus *Rhizophagus*, and decreases in those of the genus *Scutellospora* under agricultural land use accounted for the largest difference in AMF β-diversity between the agricultural land-use types and the WO reference (see PLS axis loadings). The differences among the land-use types in soil microbiological and chemical properties were mostly due to soil alkalinization (increase in pH), reduction of SOC and increases in parameters indicative of microbial activity (C loss) (see axis loadings in Figure 4A and Table 1).

The land-use types were also clearly resolved by the axes of greatest variability in AMF α-diversity and greatest variability in microbiological and chemical soil properties (Figure 4B). Differences in AMF α-diversity were mainly due to changes in Shannon diversity (H), genus richness (#AMF), dominance (D), and PD, but less due to changes in AMF abundance in roots (%AMF). The differentiation of the AMF communities among the land uses in α-diversity originated from variability in the availabilities of N, P, and K from soil, but not SOC (see axis loadings in Figure 4B).

**FIGURE 5:**
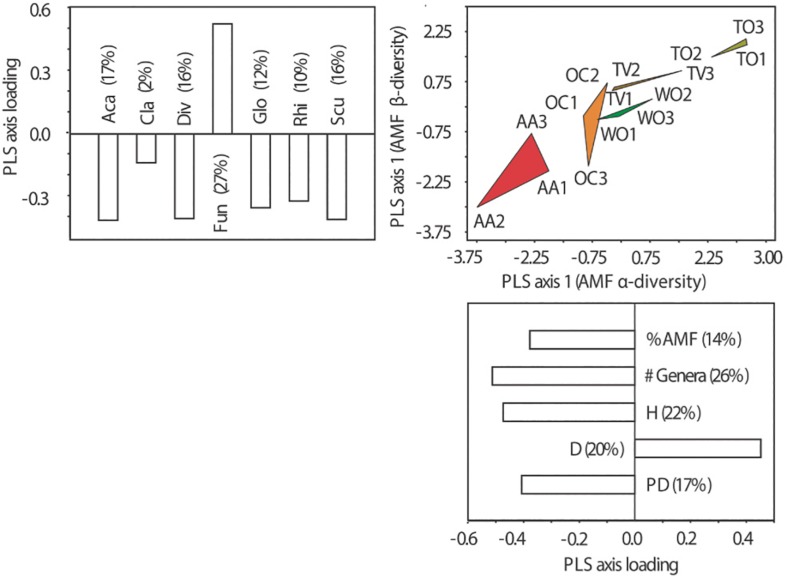
Partial least square (PLS) ordination plots separating five land-use types along the first ordination axes of α- and β-diversity of arbuscular mycorrhizal fungal (AMF) communities at the genus level. Three fields on different farms were analyzed per land-use type. The magnitude of influence (PLS axis loading) of the explanatory variables is indicated in the form of bar plots next to the ordination axes. The percentage contribution of the individual variables to the variance resolved by the respective ordination axis is indicated in brackets. See Figure 1 for land-use abbreviations. The AMF genera were: *Aca*, *Acaulospora* Gerd. and Trappe; *Cla*, *Claroideoglomus* C. Walker and A. Schüßler; *Div*, *Diversispora* C. Walker and A. Schüßler; *Fun*, *Funneliformis* C. Walker and A. Schüßler; *Glo*, *Glomus* Tul. and C. Tul.; *Rhi*, *Rhizophagus* P.A. Dang; *Scut*, *Scutellospora* C. Walker and F.E. Sanders. #AMF, number of AMF genera; %AMF, percentage of root colonization by AMF; D, dominance of AMF at the genus level; H, Shannon diversity of AMF at the genus level; PD, phylogenetic diversity of AMF.

Land uses with multiyear legume-cereal forage rotations (AA and OC), on the one hand, and cultivation of woody crops (TO and TV) under high fertilizer input and frequent surface tillage (TV and TO), on the other hand, are clearly resolved by the variance in AMF α- and β-diversity in soil (Figure 5). The resolution by AMF β-diversity is mostly due to the dominance of the AMF genus *Funneliformis* under land uses associated with frequent surface tillage. All aspects of α-diversity, however, contributed to the resolution of the land-use types along the axis of greatest variability in AMF α-diversity (see axis loadings in Figure 5).

## Discussion

This comparative field study on the influences of common Mediterranean land-use types on soil C, AMF diversity and soil fertility recognized a multiyear legume-winter cereal rotation as the land-use type that is least detrimental to C sequestration, AMF abundance and diversity in comparison to woodland reference sites. This is an important advance in recent efforts to identify land-use types and agricultural practices in the Mediterranean that do not reduce the fertility of soil over the long-term (Brito et al., 2012; Kassam et al., 2012; Aguilera et al., 2013). Using a robust multi-farm comparative approach, we found that AMF abundance and community composition and structure were heavily influenced by the type of land use and probably ultimately by the agricultural practices associated with them, such as tillage. This study thus suggests that shifts among AMF communities could be used as indicators of influences of agricultural land uses and possibly the associated soil management practices on soil fertility, taking into consideration that AMF are known to be important mediators of soil fertility, C sequestration and crop nutrition and productivity (Zhang et al., 2016; Manoharan et al., 2017; Xu et al., 2017; Powell and Rillig, 2018; Hallama et al., 2019). The direction and magnitude of change in composition and structure of the AMF communities, relative to a (semi-) natural reference ecosystem, or compared among various land-use types, can provide indications about the severity and nature of the impact of different land-use types.

### Soil Chemical and Microbiological Properties and AMF Abundance and Communities Differed Among the Five Studied Land-Use Types

The 5-year forage-winter cereal rotation with 4 years of alfalfa (AA) maintained higher SOC, C/N and MBC, compared to the other agricultural land-use types and the WO reference sites. It also sustained a high AMF root infection pressure as evident from a high colonization of the roots of English ryegrass by AMF. The soil CEC, C/N ratio, and microbial carbon (MBC) content were highest in AA and WO, where members of the AMF genus *Scutellospora* prevailed. The occurrence and prevalence of *Scutellospora* coincided, however, also with a slightly higher soil pH in WO and AA than the other agricultural land-use types. In TO and TV, on the contrary, where members of the AMF genus *Funneliformis* prevailed, soil K and P levels were high, and MBC, and by inference microbial abundance were low. This may indicate that physical soil disturbance in combination with reduced C returns to soil and reduced C allocation to AMF lowers SOC and AMF abundance and is detrimental to some AMF taxa. This is a previously not much recognized aspect. Previous studies have mostly shown that AMF taxon distribution and abundance in agroecosystems relates to soil pH and tolerance to increased mineral nutrient levels and physical disturbance by tillage (Jansa et al., 2002; Oehl et al., 2005, 2017). The comparison of the effects of the different land-use types shows, furthermore, that AA via continuous vegetation cover and plant growth throughout the year, as well as infrequent tillage, must be able to sustain high AMF abundances. That AMF benefit from infrequent tillage and a high abundance and continuous presence of host plants becomes obvious when the data of the olive and wine production systems (TO and TV) are compared to those of the forage systems (AA, OC) and woodland reference sites (WO). Under the soil management intensive agricultural land-use types, TO and TV, the root colonization by AMF in English ryegrass was particularly low, in line with the notion that frequent tillage and high nutrient availabilities are detrimental to AMF abundance (Plenchette et al., 2004) and that root colonization in English ryegrass is strongly determined by present and previous presence of mycotrophic host plants (Eason et al., 1999; Morris et al., 2013; Detheridge et al., 2016). The colonization of the roots of English ryegrass by AMF was also higher in the biannual mixed oat-clover forage-winter cereal rotation (OC) than in TO and TV, probably also because characterized by a denser and continuous herbaceous vegetation cover. This dependency of AMF root colonization on the type of land use in English ryegrass together with this grass’ wide occurrence in agroecosystems (Fuhrer, 2003) suggests English ryegrass as a suitable bioassay plant to probe the activity and abundance of AMF in observational field studies.

Our study demonstrated also that members of AMF genera, otherwise known to be either sensitive to intensive (arable) land use, or such that are in general rare, like *Acaulo-, Diversi-, and Scutellospora* (Jansa et al., 2002; Oehl et al., 2005), remain abundant under multiyear alfalfa-winter cereal forage rotation. This finding is in line with the known differential sensitivity of AMF taxa to physical soil disturbance, high mineral nutrient levels and AMF’s dependency on a continuous supply of carbohydrates from living plants (van der Heyde et al., 2017). We can thus conclude that multiyear legume-cereal forage rotation not only preserves SOC and soil microbial activity (MBC), but also sustains large AMF populations and diverse AMF communities, which has also recently been noted by Klabi et al. (2018). Since AMF are known to be functionally diverse (Maherali and Klironomos, 2007), a higher AMF diversity can be expected to allow for more different services and hence most likely increased overall crop plant benefits, including improvements to crop yield and quality and soil fertility over the long-term (Manoharan et al., 2017; Klabi et al., 2018; Powell and Rillig, 2018; Rillig et al., 2019).

The AMF communities in soil differed in their phylogenetic and genus-level taxonomic composition and structure among the different land-use types. The AMF communities of the legume-winter cereal forage rotations (AA and OC) and the tillage-intensive olive and wine production systems (TO and TV) diverged in opposite directions from those of the woodland sites. The phylogenetic and genus-level composition and structure of the AMF communities of the two types of legume-cereal forage rotation differed considerably, despite only small differences in their soil microbiological and chemical properties. This points to AMF taxon filtering by the crop during AMF community assembly as previously recorded (Ciccolini et al., 2016b) and mechanistically conceivable, since AMF are entirely dependent as obligate biotrophs on their host plants as sources of carbohydrates, the supply of which is reduced under fertile soil conditions. The AMF communities may, however, also be affected by land use-related differences in soil microbiological and chemical properties as well as site-specific differences in soil texture. To account for these confounding influences and look for consistent land-use effects, we studied the same land-use types on three different farms. Reference woodland sites were included to be able to determine trajectories and magnitudes of change in the AMF communities and soil properties.

### Agricultural Land-Use Types Are Associated With Changes in Chemical and Microbiological Soil Properties and AMF Communities

Singling out individual drivers of the observed differences in the microbiological and chemical soil fertility indicators and the AMF communities turned out as a matter of impossibility in this comparative field study. Most of the measured indicators of soil fertility and of the components of AMF diversity were highly and comparably strongly correlated to each other and associated with different land-use types. This made it necessary to link the whole set of soil microbiological and chemical properties to the whole sets of α- and β-diversity components, using partial least square (PLS) analysis, a type of analysis that can deal with such data. Considering the sign and magnitude of the axis loadings of the PLS plots allows a comparatively easy recognition of concurrent responses and effects to different land uses, as it was the case in this study with the soil chemical and microbial properties and aspects of AMF diversity. Arising patterns from PLS analyses allow recognizing possible underlying causative processes.

The PLS analysis revealed that differences in occurrence and relative abundance of members of the AMF genera *Rhizophagus* and *Scutellospora* were most strongly correlated with differences in soil C-related parameters, such as C/N, MBC, qCO_2_ and SOC. This is a novel finding, not previously reported in the literature. It suggests that different land use has altered the C-fluxes to and from the soil and C allocation to AMF. The latter would also explain differences in AMF root colonization and AMF genus occurrence and prevalence in soil. In short, this study points at land use-dependent concurrent changes in soil C and AMF communities. The PLS analysis further revealed differences in dominance of AMF genera as the most important contribution to differences in AMF α-diversity, which responded most to differences in soil pH and soil mineral nutrient contents and availabilities under the different land uses. The land uses for legume-cereal forages (AA and OC) and woody crops (TO and TV) induced divergent changes to the soil properties and AMF α-diversity with reference to the woodland (WO) control sites. We may, in fact, recognize a land use intensity gradient as previously reported in studies on AMF communities in different farming and cropping systems (Oehl et al., 2003; Lumini et al., 2010). The correlation of the α- and β-diversity datasets showed that overdominance of members of the genus *Funneliformis* influenced both α- and β-diversity. Overdominance of *Funneliformis* reduced the richness and compositional similarity of the AMF communities. All in all, we conclude from involvement of always several response parameters to a clear pattern of difference that land use impacts on soil fertility express themselves as *multivariate response syndromes* rather than changes in only few parameters.

### Possible Trait- and Life History-Related Explanations for Shifts in AMF Occurrence and Abundance

In absence of *in situ* studies on the functioning of AMF communities in agroecosystems, we can at present only speculate about the functional implications that loss of AMF taxa due to intensive agricultural land use may have (Ryan and Graham, 2018; Rillig et al., 2019; Ryan et al., 2019). Gigasporaceae, such as *Scutellospora* spp., are reported to be particularly important for plant mineral nutrient uptake (Maherali and Klironomos, 2007), while AMF richness and diversity are known to promote the productivity of grasslands through increased soil resource use by functional niche differentiation (van der Heijden et al., 1998; Wang et al., 2019). However, since crop plants are cultivated in fertile soil as single, or low-diversity mixtures, AMF diversity may overall take a less important role in their mineral nutrition, which is less compromised by competition for nutrients. Yet, high AMF diversity may still be important for not directly yield-related plant benefits, such as resistance against and tolerance of abiotic and biotic stress as well as ecosystem services, such as soil structure formation and prevention of nutrient leaching, i.e., soil health and fertility over the long-term (Jeffries et al., 2003; Powell and Rillig, 2018; Rillig et al., 2019). It is thus highly probable that diverse AMF communities are contributing to soil fertility, plant health, and hence an ecologically sustainable crop plant production in general (Mäder et al., 2002; Jeffries et al., 2003; Manoharan et al., 2017; Powell and Rillig, 2018).

In this study, members of the genus *Funneliformis* were the most abundant AMF across all land-use types. Forty-four percent of the recovered 18S rRNA gene sequences from soil were assigned to this genus. Species of this genus are known to have short life cycles (Stahl and Christensen, 1991; Oehl et al., 2009; Rosendahl et al., 2009) that may reduce their sensitivity to discontinuous plant presence and disruption of the extraradical mycelia by frequent tillage in TO and TV and thus explain their persistence and dominance under these land uses. Members of the genus *Funneliformis* may, however, also be efficient and thus competitive C scavengers when plants allocate less C to AMF under conditions of relatively low N and high K and P bioavailability in soil (Treseder, 2004; Johnson, 2010). This and their additional apparent preference for neutral to alkaline agricultural soils (Stahl and Christensen, 1991; Rosendahl et al., 2009) are alternative explanations for their relative abundance in TO and TV.

Members of the genus *Scutellospora* were only abundant in AA and WO, but not under other land-use types (OC, TO, and TV), suggesting that they may depend on continuous presence of host plants or suffer from physical disruption of their extraradical mycelia by frequent tillage under these land-use types. Members of Gigasporaceae, the family to which the genus *Scutellospora* belongs, are, in fact, known to fail to reconnect their extraradical hyphae once they are severed, rendering them particularly sensitive to physical soil disturbance (De La Providencia et al., 2005). Members of the genus *Scutellospora* must thus chiefly perennate as spores in tilled soils, while having their main growth habitat below the tillage layer under arable land use (Oehl et al., 2005). Moreover, Gigasporaceae are known to show preferences for sandy soils, to develop more hyphae in soil than roots (Maherali and Klironomos, 2007), and to drain more photosynthates from their host plants than other AMF taxa (Lerat et al., 2003; Maherali and Klironomos, 2012). The latter could indicate that Gigasporaceae have large demands of photosynthates, which alfalfa may be best able to provide all over the year and probably, particularly, under conditions of reduced mineral nutrient availability and hence shoot growth during drought periods.

Members of the AMF genus *Claroideoglomus* were only recovered from the soil of the biannual oat-clover mixture (OC) and the woodland (WO) reference sites. This may indicate that these AMF depend on dense vegetation cover like *Scutellospora* spp., but by other plants than alfalfa. Since the AMF community composition and structure were different between AA and OC, our study, in fact, may indicate that alfalfa and berseem clover could rely on different AMF. That different legume species and species abundances select for AMF communities differing in composition, structure and abundance has recently also been demonstrated experimentally (Xiao et al., 2019). Reduced AMF growth as a consequence of C limitation in absence of a dense vegetation may further explain why members of the genus *Claroideoglomus* were only infrequently recovered under the studied agricultural land-use types. Another possibility is that *Claroideoglomus* spp. are deep soil dwellers (Sosa-Hernández et al., 2018) and hence not found when soil samples are taken from only the plowed soil layer.

### Possible Methodological Biases in the Analysis of the AMF Communities From Soil

The clone library sequencing to characterize the AMF communities was shallow. Nevertheless, the accumulation curves at genus resolution reached saturation for four of five land-use types. The employed 18S rRNA marker, widely use in community studies on AMF is furthermore controversial for analyses at the species level for some lineages of AMF (Krüger et al., 2012; Bruns and Taylor, 2016). This was one reason to focus the analysis of the AMF communities at genus level. An additional reason was that trait information for AMF is only available for this level (Hart and Reader, 2002; Maherali and Klironomos, 2007). We focused our discussion and interpretation on the most solid part of our community dataset, which is AMF community difference among sites (ß-diversity) at the genus level, known to be robust to limited community sampling (Pos et al., 2014). The robustness of the genus level analyses was also evident from the comparison with the phylogenetic distances. Further studies should cover a larger geographical area, use more resolving markers and sample the community more thoroughly to confirm and generalize the findings of this study.

Members of the genus *Funneliformis* could, however, also just appear dominant in soil purely due to methodological analytical reasons. This, because the DNA from their big and enduring spores and sporocarps may well dominate over the DNA from extraradical hyphae and hence bias the structure of AMF communities with respect to plant nutritionally and soil structure-relevant hyphal abundance. On the contrary, the relative abundances of members of the AMF genera *Glomus* and *Rhizophagus* may just have appeared low, because their nucleus-rich vesicles and a large proportion of their hyphal biomass are formed in the roots and not the soil in which we recorded the AMF communities (Clapp et al., 1995; Hempel et al., 2007; Maherali and Klironomos, 2007; Balestrini et al., 2010). The uncommonly high abundance of members of the AMF genus *Scutellospora* based on the number of DNA sequences could further, as well, just have resulted from DNA of spores persisting in soil as those of members of the genus *Funneliformis*, hence, also not be indicative for the ecologically relevant hyphal abundance in soil. In short, we cannot know the magnitude of analytical bias in AMF community studies, unless AMF occurrence and abundance are simultaneously determined in roots and soil (Clapp et al., 1995; Hempel et al., 2007; Balestrini et al., 2010). Differential occurrence and abundance of several taxa suggests, however, that diversity in land uses on the same farm may at least maintain AMF diversity at the landscape scale.

## Conclusion

This comparative study on the effects of four typical Mediterranean land uses on indicators of soil fertility, and abundance, composition and structure of soil-indigenous AMF communities found multiyear forage-winter cereal rotation as the land-use type that best conserves SOC, AMF abundance and AMF taxa which are sensitive to intensive agricultural soil management. It appears that discontinuous and sparse vegetation cover and physical soil disturbance as a consequence of frequent soil tillage associated with intensive agricultural land use impair the sequestration of C in soil and growth and taxon persistence of AMF. Differences in AMF community composition and structure were more strongly related to land use and less to differences in soil texture than differences in chemical and microbiological soil properties, suggesting they could be used as robust indicators of impacts of agricultural land use on soil fertility and health.

## Data Availability Statement

The datasets generated for this study can be found in the European Molecular Biology Laboratory (accession numbers LN714838-LN715116).

## Author Contributions

EP and LE designed the study. EP carried out the fieldwork and collected the data. HG and EP performed the phylogenetic and statistical analyses, and interpreted the data. HG, EP, VC, and LE wrote and revised the manuscript.

## Conflict of Interest

VC is employed by SCL Italia SpA. The remaining authors declare that the research was conducted in the absence of any commercial or financial relationships that could be construed as a potential conflict of interest.
