# Supplementary material for: Forage Rotations Conserve Diversity of Arbuscular Mycorrhizal Fungi and Soil Fertility
Source: Front Microbiol. 2020 Jan 9;10:2969. doi: 10.3389/fmicb.2019.02969 (PMC6962183; doi:10.3389/fmicb.2019.02969)
Supplement: Supplementary file 1 [file Data_Sheet_1.PDF]

SUPPLEMENTARY MATERIAL

MATERIALS AND METHODS

TEXT S1 | Crop management practices

In the multiyear alfalfa (*Medicago sativa* L.)-winter cereal forage rotations (AA), alfalfa is fertilized with 200 kg ha^{-1} quaternary fertilizer (N-P-K-S: 15-13-13-12.5) and sown in autumn. During the four years of cultivation, alfalfa is cut twice, once in early spring and once in summer for hay making. The late summer and autumn regrowth are grassed for at most two months by 50 sheep per hectare, which leads to an input of about 1.5 t ha^{-1} of manure, corresponding to 20 kg N ha^{-1}, 10 kg P ha^{-1} and 20 kg K ha^{-1}. At the end of the fourth year, the crop is terminated in autumn by ploughing to 40 cm soil depth. After application of 150 kg ha^{-1} monoammonium phosphate fertilizer (N-P: 18-46) and disk harrowing, the winter-cereal {durum wheat [*Triticum turgidum* L. *subsp. durum* (Desf.) Husn.] or triticale (×*Triticosecale* Wittm. ex A. Camus)} is sown. No pesticides are used.

In the biannual oat (*Avena sativa* L.)-berseem clover (*Trifolium alexandrinum* L.) mixture-winter cereal forage rotations (OC), the oat-clover mixture is sown in late summer and fertilized with 16 t ha^{-1} of sheep manure (corresponding to about 120 kg N ha^{-1} 12 kg P ha^{-1} and 240 kg K ha^{-1}), and 100 kg ha^{-1} of monoammonium phosphate. The crop is grazed by sheep (50 animals per hectare) during autumn and winter and cut for hay making in May after which the regrowth is grazed over autumn and winter. The crop is terminated at the end of the second year by ploughing to 40 cm soil depth. The seed bed for the winter-cereal (durum wheat or triticale) is prepared by disk harrowing. Nitrogen fertilizer at a rate of 33.5 kg N ha^{-1} is applied at tillering in the form of ammonium nitrate. No pesticides are used.

The olive orchards were set up with *Olea europea* L. cvs Leccino, Frantoiana, Moraiolo, and Pendolino (200 plants ha^{-1} in a 7 x 7 m arrangement) in the 1960s and are fertilized in late summer with 100 t ha^{-1} of sheep manure, corresponding to about 700 kg N ha^{-1}, 70 kg P ha^{-1} and 1400 kg K ha^{-1}, incorporated to a soil depth of 15 cm by disk harrowing. Then, an Italian sainfoin (*Hedysarum coronarium* L.) - berseem clover mixture is sown as cover crop. Pruned branches are burnt locally and insecticides and fungicides used, according to an integrated management scheme.

The vineyards were planted with *Vitis vinifera* L. cv. Sangiovese (3,333 stocks ha^{-1}) in 1997. An organo-mineral fertilizer (N-P-K: 10-5-14.5) that also contains magnesium, sulphur, boron, iron, and zinc is incorporated at a rate of 200 kg ha^{-1} into the soil every April by chisel and disk harrowing. Additional surface tillage is used to control weeds in June and July. Pruned branches are burnt locally and fungicides used according to an integrated management scheme.

TEXT S2 | Summary of raw sequence data

Two hundred and seventy nine new sequences of arbuscular mycorrhizal fungi (AMF) were obtained and phylogenetically assigned to seven AMF genera: *Acaulospora*, *Claroideoglomus*, *Diversispora*, *Funneliformis*, *Glomus*, *Rhizophagus* and *Scutellospora* (**Figure 1, Table S3**), based on a ML phylogenetic analysis with the phylogenetically closest representative sequence(s) of virtual taxa (VT) from the Maarj*AM* database (**Figures 1** and **S2**). About 10% of all generated sequences had to be excluded from the analyses because of either poor quality, or non-AMF origin (data not shown). These genera belong to five AMF families, and the orders Glomerales and Diversisporales. The abundance ranking according to the number of recovered sequences at family level was: Glomeraceae (69.9%) > Gigasporaceae (26.5%) > Claroideoglomeraceae (2.2%) > Acaulosporaceae (1.1%) > Diversisporaceae (0.4%). Fourteen 18S rRNA sequence types were identical or nearly identical to VT in the MaarjAM database, including some of the most abundant sequence types (**Figures 1, Figure S2** and **Table S3**). One sequence type affiliated to the genus *Acaulospora*, six affiliated to *Funneliformis*, and one affiliated to *Scutellospora* do not have any similar VT reference sequence (**Table S3**) and must thus be AMF taxa that are not yet included in the Maarj*AM* reference database.

TABLES

TABLE S1 | Geographical coordinates of the sampled fields under five different land-use types on three farms in the municipality of Manciano (Tuscany, Central Italy). The land-use types were: AA, five-year rotations with four years of alfalfa (*Medicago sativa* L.) and one year of winter cereal [durum wheat (*Triticum turgidum* L. *subsp. durum* (Desf.) Husn. or triticale (× *Triticosecale* Wittm. ex A. Camus)]; OC, three-year rotations with two years of oat (*Avena sativa* L.) - berseem clover (*Trifolium alexandrinum* L.) mixture and one year of winter cereal (durum wheat or triticale); TO, tilled olive orchards (*Olea europea* L.); TV, tilled vineyards (*Vitis vinifera* L.); WO, woodlands dominated by *Quercus cerris* L. and *Fraxinus ornus* L. All land-use types had been in place on the same field for more than 15 years. See **FIGURE S1** for a satellite image showing the sampling sites.

Farm	Land-use type	Field ID	Latitude	Longitude
1	AA	AA1	42°31'27.28"N	11°30'21.17"E
	OC	OC1	42°31'27.50"N	11°30'33.47"E
	TO	TO1	42°31'24.46"N	11°30'35.17"E
	TV	TV1	42°31'20.74"N	11°30'45.54"E
	WO	WO1	42°31'28.13"N	11°30'23.44"E
2	AA	AA2	42°32'13.72"N	11°26'31.29"E
	OC	OC2	42°32'31.43"N	11°27'6.18"E
	TO	TO2	42°32'21.85"N	11°26'40.72"E
	TV	TV2	42°31'46.10"N	11°26'23.56"E
	WO	WO2	42°31'58.38"N	11°26'37.95"E
3	AA	AA3	42°32'2.17"N	11°27'47.28"E
	OC	OC3	42°32'18.01"N	11°27'46.97"E
	TO	TO3	42°32'10.51"N	11°27'47.14"E
	TV	TV3	42°32'6.09"N	11°27'36.50"E
	WO	WO3	42°31'58.27"N	11°27'49.00"E

TABLE S2 | Number of partial nuclear 18S rRNA gene sequences of seven genera of arbuscular mycorrhizal fungi (phylum Glomeromycota) recovered from fifteen clone libraries, representing three replicate composite soil samples of five land-use types in three farms in the municipality of Manciano (Tuscany, central Italy): AA, five-year rotations with four years of alfalfa (*Medicago sativa* L.) and one year of a winter cereal [durum wheat (*Triticum turgidum* L. *subsp. durum* (Desf.) Husn. or triticale (× *Triticosecale* Wittm. ex A. Camus)]; OC, three-year rotations with two years of oat (*Avena sativa* L.) - berseem clover (*Trifolium alexandrinum* L.) mixture and one year of winter cereal (durum wheat or triticale); TO, tilled olive orchards (*Olea europea* L.); TV, tilled vineyards (*Vitis vinifera* L.); WO, woodlands dominated by *Quercus cerris* L. and *Fraxinus ornus* L.

Land-use type	Replicate	*Acaulospora*	*Claroideoglomus*	*Diversispora*	*Funneliformis*	*Glomus*	*Rhizophagus*[1]	*Scutellospora*	Total	
AA	1	2	0	0	4	0	4	11	21	61
	2	1	0	1	5	1	3	8	19	
	3	0	0	0	5	7	1	8	21	
OC	1	0	1	0	8	2	12	0	23	62
	2	0	0	0	12	5	2	0	19	
	3	0	1	0	1	13	5	0	20	
TO	1	0	0	0	13	0	0	0	13	41
	2	0	0	0	12	0	1	0	13	
	3	0	0	0	15	0	0	0	15	
TV	1	0	0	0	10	0	7	2	19	54
	2	0	0	0	10	0	3	4	17	
	3	0	0	0	12	0	6	0	18	
WO	1	0	2	0	5	0	0	16	23	61
	2	0	0	0	6	0	0	12	18	
	3	0	2	0	5	0	0	13	20	
	Total	3	6	1	123	28	44	74	279	279

[1]Please note that the new genus name of *Rhizophagus* P.A. Dang is now *Rhizoglomus* Sieverd., G.A. Silva & Oehl (Sieverding et al., 2015). We decided to stick to the old name, however, because the majority of sequence accessions in the public databases are still annotated with this name, or the earlier name of this genus, *Glomus* Tul. & C. Tul..c

Sieverding, E., da Silva, G.A., Berndt, R., Oehl, F. (2015). *Rhizoglomus*, a new genus of the Glomeraceae. *Mycotaxon* 129, 373-386. doi: 10.5248/129.373

TABLE S3 | List of the representative sequences used to infer the maximum likelihood tree (FIGURE 1). Sequences identical in the aligned positions considered for the phylogenetic analysis are listed together with the *Virtual Taxon* (VTX) of the MaarjAM database (Öpik et al. 2010) with >97% sequence identity. The genus affiliations are indicated in the last column.

Representative sequence	Other sequences	Virtual Taxon	Genus affiliation
LN715044		VTX00108	*Rhizophagus*[1]
LN715043		VTX00108	*Rhizophagus*
LN714892	LN714927, LN714925	VTX00105	*Rhizophagus*
LN715013	LN715014	VTX00113	*Rhizophagus*
LN714998	LN714999	VTX00113	*Rhizophagus*
LN715089		VTX00113	*Rhizophagus*
LN715092		VTX00113	*Rhizophagus*
LN715054		VTX00113	*Rhizophagus*
LN714994	LN714995	VTX00113	*Rhizophagus*
LN715011	LN715012	VTX00113	*Rhizophagus*
LN715035		VTX00113	*Rhizophagus*
LN715109		VTX00114	*Rhizophagus*
LN714994	LN714995	VTX00114	*Rhizophagus*
LN714840		VTX00114	*Rhizophagus*
LN714891	LN714895	VTX00114	*Rhizophagus*
LN715069		VTX00114	*Rhizophagus*
LN715010		VTX00114	*Rhizophagus*
LN715009		VTX00114	*Rhizophagus*
LN715007	LN715008, LN714923, LN714926	VTX00114	*Rhizophagus*
LN715091		VTX00363	*Rhizophagus*
LN715016	LN715017	VTX00363	*Rhizophagus*
LN714838	LN714905, LN714904, LN714839	VTX00067	*Funneliformis*
LN714899	LN714914, LN714932, LN714939, LN714978	VTX00067	*Funneliformis*
LN714844	LN714853, LN714848, LN714865, LN714861, LN714857, LN714881, LN714877, LN714869, LN714921	VTX00067	*Funneliformis*
LN714841	LN714850, LN714849, LN714845, LN714862, LN714858, LN714854, LN714878, LN714874, LN714873, LN714870, LN714919	VTX00067	*Funneliformis*
LN714918	LN714930	VTX00067	*Funneliformis*
LN714842	LN714996, LN714851, LN714846, LN714863, LN714859, LN714855, LN714879, LN714875, LN714871, LN714867, LN714903, LN714897, LN714888, LN714924, LN715116, LN714937, LN714935, LN714928, LN714938, LN714966, LN714977	VTX00067	*Funneliformis*
LN714997		VTX00067	*Funneliformis*
LN715020	LN715021	VTX00067	*Funneliformis*
LN714997		VTX00067	*Funneliformis*
LN715020	LN715021	VTX00067	*Funneliformis*
LN715003		VTX00067	*Funneliformis*
LN714900	LN714915, LN714933	VTX00067	*Funneliformis*
LN714917	LN714929	VTX00067	*Funneliformis*
LN714906	LN714931	VTX00067	*Funneliformis*
LN715030	LN715031	VTX00067	*Funneliformis*
LN715006	LN715027	VTX00067	*Funneliformis*
LN714993		VTX00067	*Funneliformis*
LN714902	LN714936	VTX00067	*Funneliformis*
LN714901	LN714916, LN714934	VTX00067	*Funneliformis*
LN715018	LN715019	VTX00067	*Funneliformis*
LN714843	LN714852, LN714864, LN714860, LN714880, LN714876, LN714872, LN714868, LN714920, LN714847, LN714856	VTX00067	*Funneliformis*

Representative sequence	Other sequences	Virtual taxon	Genus affiliation
LN715042	LN715115	VTX00067	*Funneliformis*
LN714943	LN714944, LN714970, LN714969, LN714968, LN714967, LN714982, LN714981	VTX00065	*Funneliformis*
LN715058	LN715062, LN715082, LN715078, LN715102, LN715098	VTX00065	*Funneliformis*
LN715057	LN715061, LN715081, LN715077, LN715101, LN715097	VTX00065	*Funneliformis*
LN715090		none	*Funneliformis*
LN714945	LN714953, LN714983	none	*Funneliformis*
LN715029		none	*Funneliformis*
LN715028		none	*Funneliformis*
LN715004	LN715005	none	*Funneliformis*
LN715047		none	*Funneliformis*
LN715039	LN715112	VTX00342	*Glomus*
LN715053		VTX00419	*Glomus*
LN715038	LN715111	VTX00419	*Glomus*
LN715046	LN715093, LN715110	VTX00419	*Glomus*
LN715050	LN715051	VTX00419	*Glomus*
LN715002		VTX00419	*Glomus*
LN715022		VTX00419	*Glomus*
LN715036		VTX00419	*Glomus*
LN715108		VTX00418	*Glomus*
LN715037		VTX00418	*Glomus*
LN715000	LN715001, LN715033, LN715032, LN715015	VTX00156	*Glomus*
LN715034		VTX00156	*Glomus*
LN715025	LN715114, LN715105, LN715041	VTX00156	*Glomus*
LN715023	LN715024	VTX00156	*Glomus*
LN715052		VTX00156	*Glomus*
LN715049		VTX00156	*Glomus*
LN715048		VTX00156	*Glomus*
LN715040	LN715113	VTX00156	*Glomus*
LN714940	LN714979	VTX00193	*Claroideoglomus*
LN714942	LN714980	VTX00193	*Claroideoglomus*
LN715026		VTX00193	*Claroideoglomus*
LN715045		VTX00193	*Claroideoglomus*
LN715063	LN715064, LN715084, LN715083, LN715104, LN715103	none	*Scutellospora*
LN714882	LN714887, LN714886, LN714885, LN714884, LN714883, LN714991, LN714965, LN714964, LN714963, LN714962, LN714961, LN714960, LN714959, LN714958, LN714990, LN714976, LN714975, LN714974, LN714973, LN714972, LN714971	VTX00041	*Scutellospora*
LN714896	LN714898, LN714913, LN714912, LN714911, LN714910	VTX00254	*Scutellospora*
LN714941	LN714948, LN714947, LN714946, LN714954, LN714987, LN714986, LN714985, LN714984	VTX00039	*Scutellospora*
LN714949	LN714992, LN714952, LN714951, LN714950, LN714957, LN714956, LN714955, LN714989, LN714988	VTX00039	*Scutellospora*
LN715072		VTX00052	*Scutellospora*
LN715070		VTX00052	*Scutellospora*
LN715071		VTX00052	*Scutellospora*
LN715055	LN715067, LN715060, LN715056, LN715087, LN715080, LN715076, LN715107, LN715100, LN715096, LN715066, LN715059, LN715094, LN715085, L N715079, LN715106, LN715099, LN715095	VTX00052	*Scutellospora*
LN715073		VTX00049	*Scutellospora*

Representative sequence	Other sequences	Virtual taxon	Genus affiliation
LN715088		VTX00263	*Diversispora*
LN715075		VTX00026	*Acaulospora*
LN715074		VTX00030	*Acaulospora*
LN715086		none	*Acaulospora*

[1]Please note that the new genus name of *Rhizophagus* P.A. Dang is now *Rhizoglomus* Sieverd., G.A. Silva & Oehl (Sieverding et al., 2015). We decided to stick to the old name, however, because the majority of sequence accessions in the public databases are still annotated with this name, or the earlier name of this genus, *Glomus* Tul. & C. Tul..c

Sieverding, E., da Silva, G. A., Berndt, R., Oehl, F. (2015). *Rhizoglomus*, a new genus of the Glomeraceae. *Mycotaxon* 129, 373-386. doi: 10.5248/129.373

FIGURES

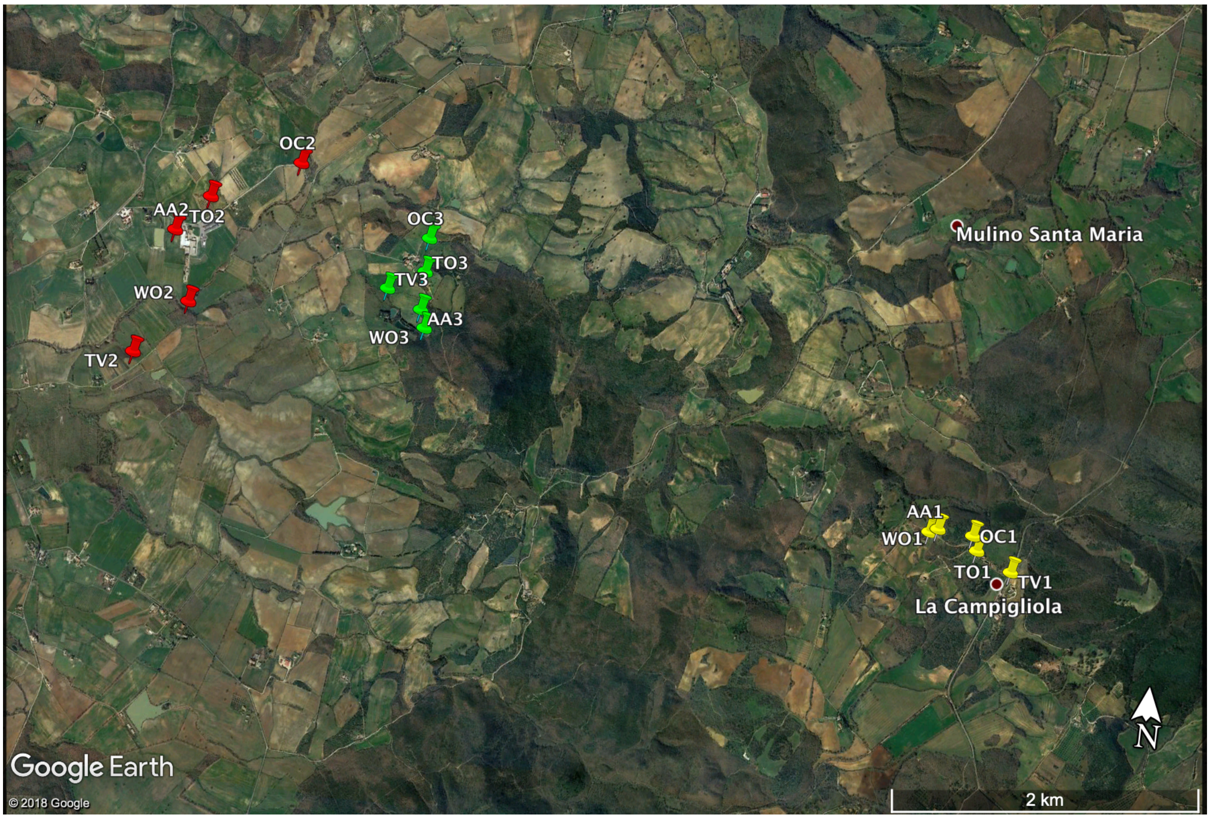

FIGURE S1 | Google satellite image showing three farms (Farm 1, Farm 2, Farm 3) in the municipality of Manciano (Tuscany, central Italy), in which five land-use types were sampled: AA, five year rotations with four years of alfalfa (*Medicago sativa* L.) and one year of winter cereal [durum wheat (*Triticum turgidum* L. *subsp. durum* (Desf.) Husn. or triticale (× *Triticosecale* Wittm. ex A. Camus)]; OC, three year rotations with two years of oat (*Avena sativa* L.) - berseem clover (*Trifolium alexandrinum* L.) mixture and one year of winter cereal (durum wheat or triticale); TO, tilled olive orchards (*Olea europea* L.); TV, tilled vineyards (*Vitis vinifera* L.); WO, woodlands dominated by *Quercus cerris* L. and *Fraxinus ornus* L. All land-use types had been in place on the same field for more than 15 years. See **TABLE S1** for geographical coordinates of the sampled fields.

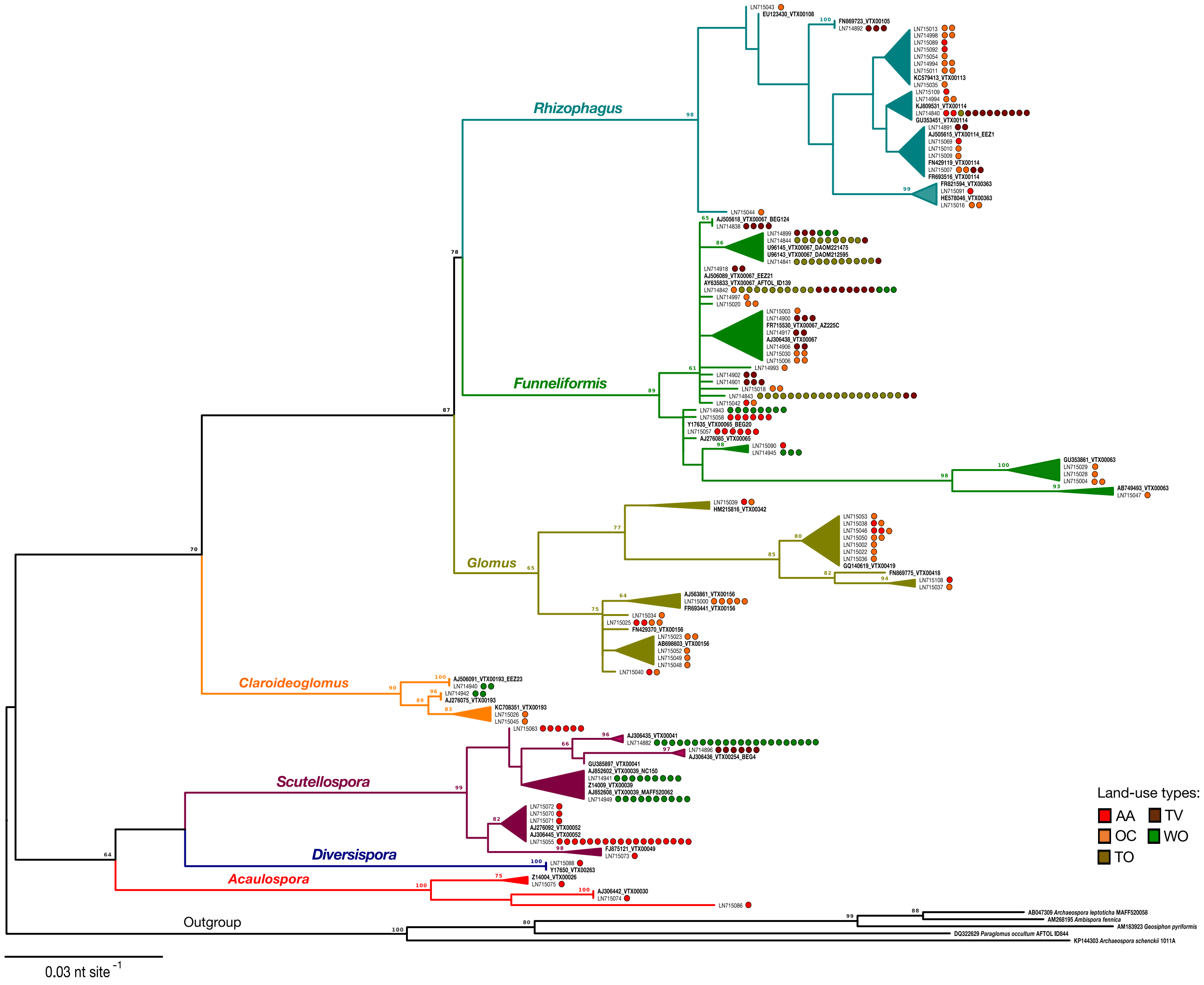

FIGURE S2 | Maximum likelihood tree to phylogenetically assign the new partial 18S rRNA gene sequences from soil to seven genera of arbuscular mycorrhiza fungi (AMF, phylum Glomeromycota) and accession sequences of *Virtual Taxa* (VT) of the MaarjAM database (Öpik et al., 2010). All new sequences and phylogenetically most related reference sequences of VT are shown with their accession number. Sequence origin from the five land-use types is indicated with coloured dots. See legend to **FIGURE 2** for further details. The tree is identical to that shown in **FIGURE 1**.

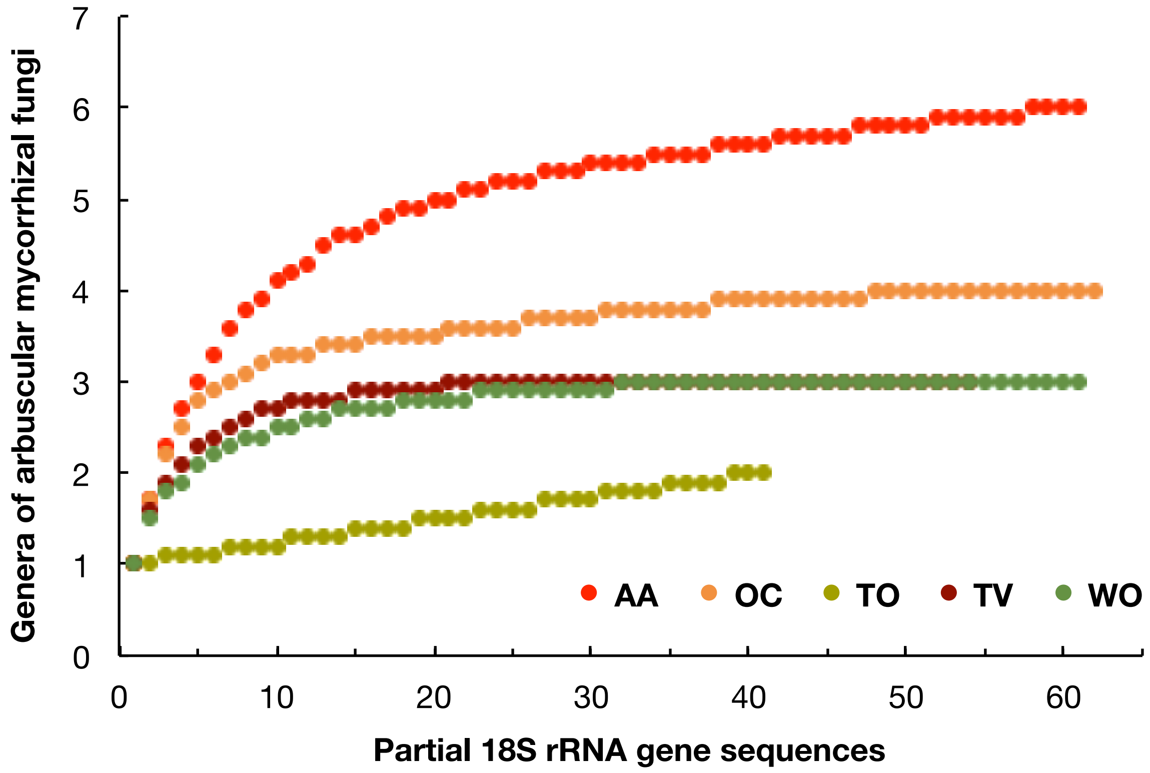

FIGURE S3 | Rarefaction curves showing the relationship between the number of genera of arbuscular mycorrhizal fungi (AMF) recovered from soil of five land-use types with increasing effort of sequencing clone libraries with inserts of partial nuclear 18S rRNA gene amplicons. The effects of the land-use types on AMF richness were studied on three farms in the municipality of Manciano (Tuscany, central Italy): AA, five-year rotations with four years of alfalfa (*Medicago sativa* L.) and one year of winter cereal [durum wheat (*Triticum turgidum* L. *subsp. durum* (Desf.) Husn. or triticale (× *Triticosecale* Wittm. ex A. Camus)]; OC, three-year rotations with two years of oat (*Avena sativa* L.) - berseem clover (*Trifolium alexandrinum* L.) mixture and one year of winter cereal (durum wheat or triticale); TO, tilled olive orchards (*Olea europea* L.); TV, tilled vineyards (*Vitis vinifera* L.); WO, woodlands dominated by *Quercus cerris* L. and *Fraxinus ornus* L.

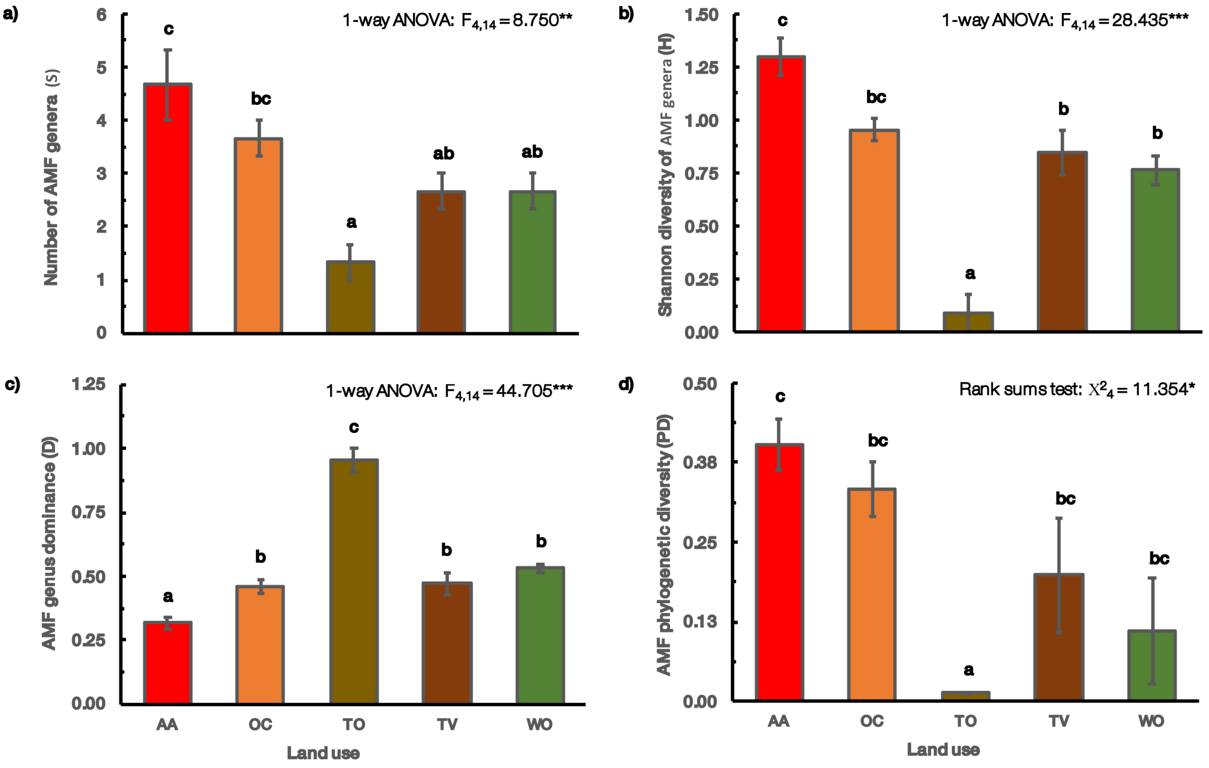

FIGURE S4 | Number of arbuscular mycorrhizal fungal (AMF) genera (S: #AMF) **(a)**, Shannon diversity (H) **(b)**, dominance (D) **(c)** of AMF genera and phylogenetic diversity (PD) **(d)** in the soil under five land-use types on three farms in the municipality of Manciano (Tuscany, central Italy): AA, five-year rotations with four years of alfalfa (*Medicago sativa* L.) and one year of winter cereal [durum wheat (*Triticum turgidum* L. *subsp. durum* (Desf.) Husn. or triticale (× *Triticosecale* Wittm. ex A. Camus)]; OC, three-year rotations with two years of oat (*Avena sativa* L.) - berseem clover (*Trifolium alexandrinum* L.) mixture and one year of winter cereal (durum wheat or triticale); TO, tilled olive orchards (*Olea europea* L.); TV, tilled vineyards (*Vitis vinifera* L.); WO, woodland dominated by *Quercus cerris* L. and *Fraxinus ornus* L. The statistical results of one-way analyses of variance (a-c) and of the Wilcoxon/Kruskal-Wallis rank sum test (d) are given on the top right of each subfigure. Different letters above the bars indicate statistically significant differences according to Tukey test (a-c) and to the Dunn nonparametric test ($P \leq 0.05$) (d).

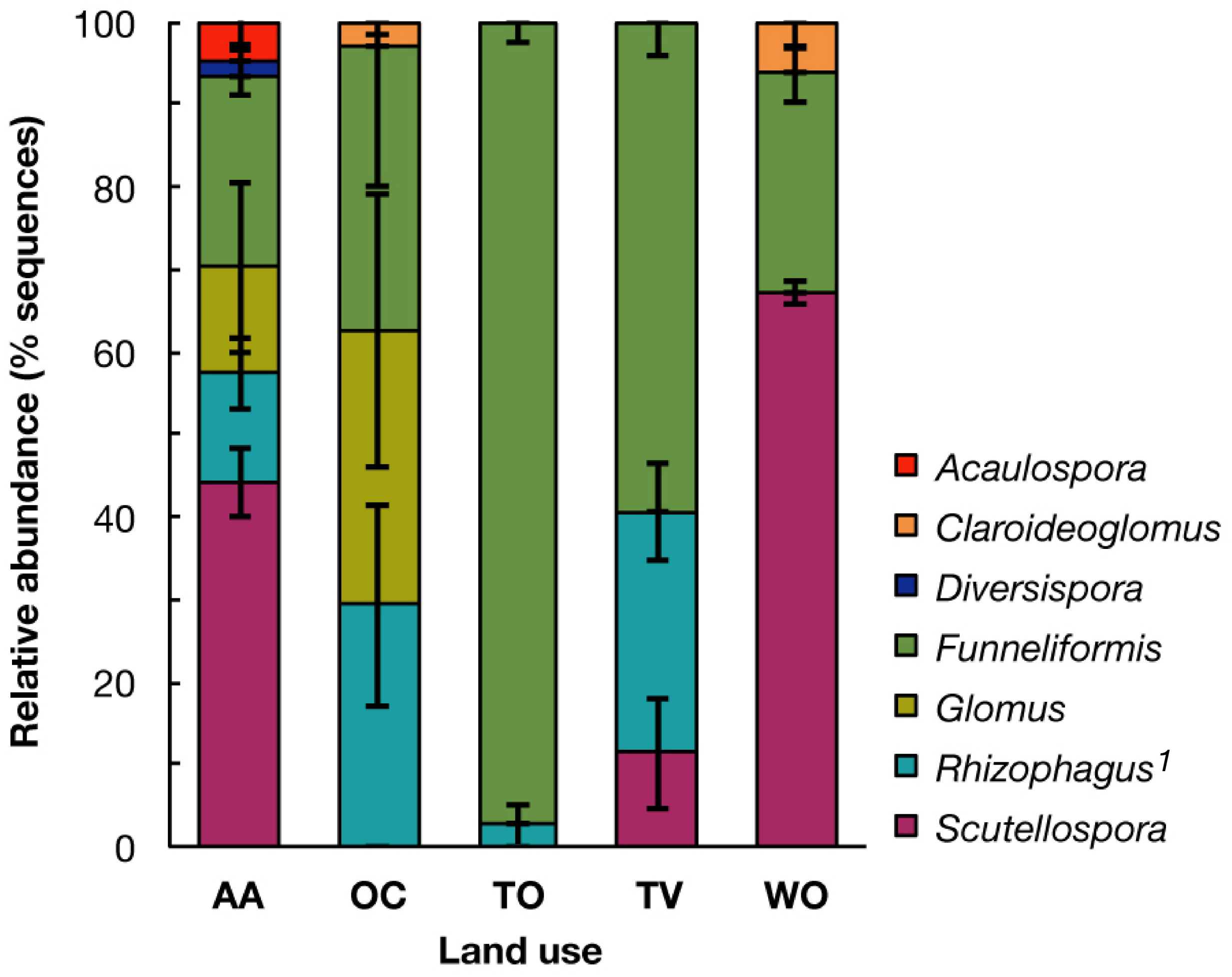

FIGURE S5 | Relative abundance of seven arbuscular mycorrhizal fungal genera (phylum Glomeromycota) in soil under five land-use types of three farms in the municipality of Manciano (Tuscany, central Italy). The relative abundances were calculated per soil sample and means ± SE of three fields on different farms per land-use type. The land uses were: AA, five-year rotations with four years of alfalfa (*Medicago sativa* L.) and one year of winter cereal [durum wheat (*Triticum turgidum* L. *subsp. durum* (Desf.) Husn. or triticale (× *Triticosecale* Wittm. ex A. Camus)]; OC, three-year rotations with two years of oat (*Avena sativa* L.) - berseem clover (*Trifolium alexandrinum* L.) mixture and one year of winter cereal (durum wheat or triticale); TO, tilled olive orchards (*Olea europea* L.); TV, tilled vineyards (*Vitis vinifera* L.); WO, woodlands dominated by *Quercus cerris* L. and *Fraxinus ornus* L..

[1]Please note that the new genus name of *Rhizophagus* P.A. Dang is now *Rhizoglomus* Sieverd., G.A. Silva & Oehl (Sieverding et al., 2015). We decided to stick to the old name, however, because the majority of sequence accessions in the public databases are still annotated with this name, or the earlier name of this genus, *Glomus* Tul. & C. Tul..c

Sieverding, E., da Silva, G. A., Berndt, R., Oehl, F. (2015). *Rhizoglomus*, a new genus of the Glomeraceae. *Mycotaxon* 129, 373-386. doi: 10.5248/129.373

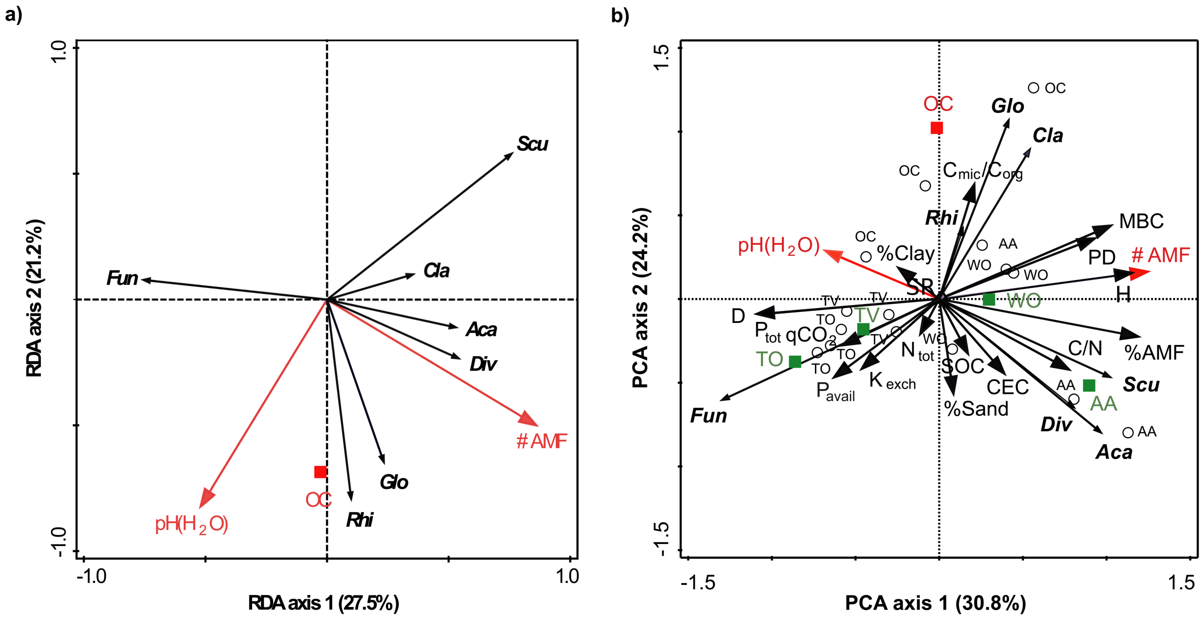

FIGURE S6 | Analysis on the effects of land use on soil microbiological and chemical properties, diversity of arbuscular mycorrhizal fungi (AMF) in soil and AMF abundance in roots of English ryegrass (*Lolium perenne* L.) **(a)** Redundancy analysis (RDA) biplot showing only those explanatory variables (red arrows and square) that are significantly correlated with AMF genus occurrence and relative abundance (black arrows). **(b)** Principle component analysis (PCA) triplot showing the degree of correlation among and relative importance of all those microbiological and chemical soil parameters, which differed significantly among land-use types, according to univariate analyses of variance (**TABLE 1**) and the recorded parameters of AMF diversity. The data were collected from replicate three s (composite samples) in three farms in the municipality of Manciano (Tuscany, central Italy). The percentage of total variance explained by the ordination axis is indicated in parentheses. AMF genera: *Aca*, *Acaulospora* Gerd. & Trappe; *Cla*, *Claroideoglomus* C. Walker & A. Schüßler; *Div*, *Diversispora* C. Walker & A. Schüßler; *Fun*, *Funneliformis* C. Walker & A. Schüßler; *Glo*, *Glomus* Tul. & C. Tul.; *Rhi*, *Rhizophagus* P.A. Dang[1]; *Scut*, *Scutellospora* C. Walker & F.E. Sanders. Land-use types: AA, five-year rotations with four years of alfalfa (*Medicago sativa* L.) and one year of winter cereal [durum wheat (*Triticum turgidum* L. *subsp. durum* (Desf.) Husn. or triticale (× *Triticosecale* Wittm. ex A. Camus)]; OC, three-year rotations with two years of oat (*Avena sativa* L.) - berseem clover (*Trifolium alexandrinum* L.) mixture and one year of winter cereal (durum wheat or triticale); TO, tilled olive orchards (*Olea europea* L.); TV, tilled vineyards (*Vitis vinifera* L.); WO, woodlands dominated by *Quercus cerris* L. and *Fraxinus ornus* L. #AMF, number of AMF genera (S); %AMF, percentage of root colonisation by AMF; %Clay, percentage of clay; C/N, carbon to nitrogen ratio; CEC, carbon exchange capacity; C_{mic}/C_{org}, ratio of microbial to organic carbon; D, dominance of AMF at the genus level; MBC, microbial carbon; H, Shannon diversity of AMF at the genus level; K_{exch}, exchangeable potassium; N_{tot}, total nitrogen; PD, phylogenetic diversity of AMF; P_{avail}, bioavailable P; P_{tot}, total P; qCO_2, metabolic quotient; %Sand, percentage of sand; SOC, soil organic carbon; SR, soil respiration. See Materials and Methods for further details.

[1]Please note that the new genus name of *Rhizophagus* P.A. Dang is now *Rhizoglomus* Sieverd., G.A. Silva & Oehl (Sieverding et al., 2015). We decided to stick to the old name, however, because the majority of sequence accessions in the public databases are still annotated with this name, or the earlier name of this genus, *Glomus* Tul. & C. Tul..c

Sieverding, E., da Silva, G. A., Berndt, R., Oehl, F. (2015). *Rhizoglomus*, a new genus of the Glomeraceae. *Mycotaxon* 129, 373-386. doi: 10.5248/129.373

R CODES

CODE S1 | Command line codes used in the software package *mothur*:

For calculating *UniFrac distances* and *Faith's Phylogenetic Diversity (PD)* indices per study site:
Construction of a count table, showing the number of representatives of the representative sequences found at each study site:
./*mothur* "#make.table(group=group_file_sites.txt, name=name_file.txt)"
Calculation of the weighted UniFrac distances of the AMF communities among all sites:
./*mothur* "#unifrac.weighted(tree=RAxML_best_tree.nxs, count=name_file.count_table, iters=10000, random=t, distance=lt)"
Calculation of Faith's Phylogenetic Diversity (PD) per site:
./*mothur* "#phylo.diversity(tree=RAxML_best_tree.nxs, group=group_file_plot.txt, name=name_file.txt, rarefy=T, collect=T, summary=T, iters=100000, groups=all, scale=f)"

CODE S2 | Command line codes used in the software package *R*:

Indicator species analysis according to Dufrene, M. and Legendre, P. 1997. Species assemblages and indicator species: the need for a flexible asymmetrical approach. Ecol. Monogr. 67(3):345-366.
Indicator values 'd' are the product of the relative frequency and relative average abundance of each species in each group of sites per unit of interest.

```
# Loading the R package:
library(labdsv)
# Read in the relative AMF genus abundances per land use as calculated from the number sequence reads:
Rel_AMF_genus_abundance_per_land_use<- read.csv(file="/path
/Working_directory/Rel_AMF_genus_abundance_per_land_use.csv", check.names=FALSE, header=TRUE,
row.names=1, sep=",")
# Define the land use types:
Land_use_types<-c("AA","OC","TO","TV","WO")
# Running the indicator analysis:
Indicator_AMF_genera_for_land_uses<-indval(Rel_AMF_genus_abundance_per_land_use, Land_use_types,
numitr=1000)
# Getting the summary table:
Summary_table_of_indicator_AMF_genera_for_land_use_types<-
summary(Indicator_AMF_genera_for_land_uses, p=0.05, digits=2, show=p, sort=FALSE, too.many=100)
# Extracting the indicator values:
ind_val_of_AMF_genus_in_land_use_types<-Indicator_AMF_genera_for_land_uses$indval
ind_val_of_AMF_genus_in_land_use_types
# Extracting the strongest indicator AMF_genera:
AMF_genus_is_best_indicator_for_land_use_type<-Indicator_AMF_genera_for_land_uses$maxcls
```

```
AMF_genus_is_best_indicator_for_land_use_type
# Extracting the respective indicator values:
ind_value_of_AMF_genus_as_best_indicator_for_land_use<-Indicator_AMF_genera_for_land_uses$indcls
ind_value_of_AMF_genus_as_best_indicator_for_land_use
# Extracting the significance values:
p_value_for_ind_value_of_AMF_genus_for_land_use_type<-Indicator_AMF_genera_for_land_uses$pval
p_value_for_ind_value_of_AMF_genus_for_land_use_type
```
